# The Entropic Dynamics Approach to Quantum Mechanics

**DOI:** 10.3390/e21100943

**Published:** 2019-09-26

**Authors:** Ariel Caticha

**Affiliations:** Physics Department, University at Albany-SUNY, Albany, NY 12222, USA; ariel@albany.edu

**Keywords:** quantum mechanics, entropic dynamics, symplectic geometry, information geometry

## Abstract

Entropic Dynamics (ED) is a framework in which Quantum Mechanics is derived as an application of entropic methods of inference. In ED the dynamics of the probability distribution is driven by entropy subject to constraints that are codified into a quantity later identified as the phase of the wave function. The central challenge is to specify how those constraints are themselves updated. In this paper we review and extend the ED framework in several directions. A new version of ED is introduced in which particles follow smooth differentiable Brownian trajectories (as opposed to non-differentiable Brownian paths). To construct ED we make use of the fact that the space of probabilities and phases has a natural symplectic structure (i.e., it is a phase space with Hamiltonian flows and Poisson brackets). Then, using an argument based on information geometry, a metric structure is introduced. It is shown that the ED that preserves the symplectic and metric structures—which is a Hamilton-Killing flow in phase space—is the linear Schrödinger equation. These developments allow us to discuss why wave functions are complex and the connections between the superposition principle, the single-valuedness of wave functions, and the quantization of electric charges. Finally, it is observed that Hilbert spaces are not necessary ingredients in this construction. They are a clever but merely optional trick that turns out to be convenient for practical calculations.

## 1. Introduction

Quantum mechanics has been commonly regarded as a generalization of classical mechanics with an added element of indeterminism. The standard quantization recipe starts with a description in terms of the system’s classical coordinates and momenta {q,p} and then proceeds by applying a series of more or less ad hoc rules that replace the classical {q,p} by self-adjoint linear operators $\left\{\hat{q},\hat{p}\right\}$ acting on some complex Hilbert space [1]. The Hilbert space structure is given priority while the probabilistic structure is relegated to the less fundamental status of providing phenomenological rules for how to handle those mysterious physical processes called measurements. The result is a dichotomy between two separate and irreconcilable modes of wave function evolution: one is the linear and deterministic Schrödinger evolution and the other is the discontinuous and stochastic wave function collapse [2,3]. To put it bluntly, the dynamical and the probabilistic aspects of quantum theory are incompatible with each other. And furthermore, the dichotomy spreads to the interpretation of the quantum state itself [4,5,6,7,8]. It obscures the issue of whether the wave function describes the *ontic* state of the system or whether it describes an *epistemic* state about the system [9].

In the Entropic Dynamics (ED) approach these problems are resolved by placing the probabilistic aspects of QM at the forefront while the Hilbert space structure is relegated to the secondary role of a convenient calculational tool [10,11,12]. ED tackles QM as an example of entropic inference, a framework designed to handle insufficient information [13,14,15,16,17,18]. The starting point is to specify the subject matter, the ontology—are we talking about the positions of particles or the configurations of fields? Once this decision is made our inferences about these variables are driven by entropy subject to information expressed by constraints. The main effort is directed towards choosing those constraints since it is through them that the “physics” is introduced.

From the ED perspective many of the questions that seemed so urgent in other approaches are successfully evaded. For example, when quantum theory is regarded as an extension of classical mechanics any deviations from causality demand an explanation. In contrast, in the entropic approach uncertainty and probabilities are the norm. Indeterminism is just the inevitable consequence of incomplete information and no deeper explanation is needed. Instead, it is the certainty and determinism of the classical limit that require explanations. Another example of a question that has consumed an enormous effort is the problem of deriving the Born rule from a fundamental Hilbert space structure. In the ED approach this question does not arise and the burden of explanation runs in the opposite direction: how do objects such as wave functions involving complex numbers emerge in a purely probabilistic framework? Yet a third example concerns the interpretation of the wave function itself. ED offers an uncompromising and radically epistemic view of the wave function Ψ. This turns out to be extremely restrictive: in a fully epistemic interpretation there is no logical room for “quantum” probabilities obeying alternative rules of inference. Not only is the probability ${\left|\Psi \right|}^{2}$ interpreted as a state of knowledge but, in addition, the epistemic significance of the phase of the wave function must be clarified and made explicit. Furthermore, it is also required that all *updates of* Ψ, which include *both* its unitary time evolution *and* the wave function collapse during measurement, must be obtained as a consequence of entropic and Bayesian updating rules [19,20,21,22,23,24].

There is a large literature on reconstructions of quantum mechanics (see e.g., [25,26,27,28,29,30,31] and references therein) and there are several approaches based on information theory (see e.g., [32,33,34,35,36,37,38,39,40,41,42,43,44,45,46]). What distinguishes ED is a strict adherence to Bayesian and entropic methods and a central concern with the nature of time. The issue here is that any discussion of dynamics must inevitably include a notion of time but the rules for inference do not mention time—they are totally atemporal. One can make inferences about the past just as well as about the present or the future. This means that any model of dynamics based on inference must also include assumptions about time, and those assumptions must be explicitly stated. In ED “entropic” time is a book-keeping device *designed* to keep track of changes. The construction of entropic time involves several ingredients. One must introduce the notion of an ‘instant’; one must show that these instants are suitably ordered; and finally, one must define a convenient measure of the duration or interval between the successive instants. It turns out that an arrow of time is generated automatically and entropic time is intrinsically directional.

This paper contains a review of previous work on ED and extends the formalism in several new directions. In [10,11,12] the Schrödinger equation was derived as a peculiar non-dissipative diffusion in which the particles perform an irregular Brownian motion that resembles the Einstein–Smoluchowski (ES) process [47]. The trajectories are continuous and non-differentiable so their velocity is undefined. Since the expected length of the path between any two points is infinite this would be a very peculiar motion indeed. Here we exhibit a new form of ED in which the Brownian motion resembles the much smoother Oernstein–Uhlenbeck (OU) process [47]. The trajectories have finite expected lengths; they are continuous and differentiable. On the other hand, although the velocities are well defined and continuous, they are not differentiable [25,48].

We had also shown that the irregular Brownian motion at the “microscopic” or sub-quantum level was not unique. One can enhance or suppress the fluctuations while still obtaining the same emergent Schrödinger behavior at the “macroscopic” or quantum level [49,50]. A similar phenomenon is also found in the smoother ED developed here. In both the ES and the OU cases the special limiting case in which fluctuations are totally suppressed turns out to be of particular interest because the particles evolve deterministically along the smooth lines of probability flow. This means that ED includes the Bohmian or causal form of quantum mechanics [51,52,53] as a limiting case.

ED consists of the entropic updating of probabilities through information supplied by constraints. The main concern is how these constraints are chosen including, in particular, how the constraints themselves are updated. In [54] an effective criterion was found by adapting Nelson’s seminal insight that QM is a non-dissipative diffusion [55]. This amounts to updating constraints in such a way that a certain energy functional is conserved. Unfortunately, this criterion, while fully satisfactory in a non-relativistic setting, fails in curved space-times where the concept of a globally conserved energy may not exist.

The second contribution in this paper is a geometric framework for updating constraints that does not rely on the notion of a conserved energy. Our framework draws inspiration from two sources: one is the fact that QM has a rich geometrical structure [56,57,58,59,60,61,62,63,64]. The authors of [56,57,58,59,60,61,62] faced the task of unveiling geometric structures that, although well hidden, are already present in the standard QM framework. Our goal runs in the opposite direction: we impose these natural geometric structures as the foundation upon which we reconstruct the QM formalism.

The other source of inspiration is the connection between QM and information geometry [17,65,66,67,68] that was originally suggested in the work of Wootters [32]. This connection has been explored in the context of quantum statistical inference [69], in the operational description of quantum measurements [37,39], and in the reconstruction of QM [43,44]. Our previous presentation in [12] has been considerably streamlined by recognizing the central importance of symmetry principles when implemented in conjunction with concepts of information geometry.

In ED, the degrees of freedom are the probability densities ρ(x) and certain “phase” fields Φ(x) that represent the constraints that control the flow of probabilities. Thus, we are concerned not just with the “configuration” space of probabilities {ρ} but with the larger space of probabilities and phases {ρ,Φ}. The latter has a natural symplectic structure, i.e., {ρ,Φ} is a phase space. Imposing a dynamics that preserves this symplectic structure leads to Hamiltonian flows, Poisson brackets, and so much of the canonical formalism associated with mechanics. To single out the particular Hamiltonian flow that reproduces QM we extend the information geometry of the configuration space {ρ} to the full phase space. This is achieved by imposing a symmetry that is natural in a probabilistic setting: we extend the well-known spherically symmetric information geometry of the space {ρ} to the full phase space {ρ,Φ}. This construction yields a derivation of the Fubini–Study metric. A welcome by-product is that the joint presence of a symplectic and a metric structure leads to a complex structure. This is the reason QM involves complex numbers.

The dynamics that preserves the metric structure is a Killing flow. We propose that the desired geometric criterion for updating constraints is a dynamics that preserves both the symplectic and the metric structures. Thus, in the final step of our reconstruction of QM we show that the Hamiltonians that generate Hamiltonian-Killing flows lead to an entropic dynamics described by the linear Schrödinger equation.

We conclude with some comments exploring various aspects of the ED formalism. We show that despite the arrow of entropic time, the resulting ED is symmetric under time reversal. We discuss the connections between linearity, the superposition principle, the single-valuedness of wave functions, and the quantization of charge. We also discuss the classical limit and the Bohmian limit in which fluctuations are suppressed and particles follow deterministic trajectories. Finally, we discuss the introduction of Hilbert spaces. We argue that while strictly unnecessary in principle, Hilbert spaces are extremely convenient for calculational purposes.

This paper focuses on the derivation of the Schrödinger equation but the ED approach has been applied to a variety of other topics in quantum theory. These include: the quantum measurement problem [70,71]; momentum and uncertainty relations [50,72] (see also [73,74,75,76]); the Bohmian limit [49,50] and the classical limit [77]; extensions to curved spaces [78]; to relativistic fields [79,80]; and the ED of spin [81].

## 2. The ED of Short Steps

We deal with *N* particles living in a flat 3-dimensional space **X** with metric $\delta_{ab}$. For *N* particles the configuration space is $X_{N}=X\times \ldots \times X$. We assume that the particles have definite positions $x_{n}^{a}$ and it is their unknown values that we wish to infer [82]. (The index n=1,…,N denotes the particle and a=1,2,3 the spatial coordinates.)

In ED positions play a very special role: they define the ontic state of the system. This is in contradiction with the standard Copenhagen notion that quantum particles acquire definite positions only as a result of a measurement. For example, in the ED description of the double slit experiment the particle definitely goes through one slit or the other but one might not know which. The wave function, on the other hand, is a purely epistemic notion and, as it turns out, all other quantities, such as energy or momentum, are epistemic too. They do not reflect properties of the particles but properties of the wave function [70,71,72].

Having identified the microstates $x\in X_{N}$ we tackle the dynamics. The main dynamical assumption is that the particles follow trajectories that are continuous. This represents an enormous simplification because it implies that a generic motion can be analyzed as the accumulation of many infinitesimally short steps. Therefore, the first task is to find the transition probability $P(x^{\prime }|x)$ for a short step from an initial *x* to an unknown neighboring $x^{\prime }$ and only later we will determine how such short steps accumulate to yield a finite displacement.

The probability $P(x^{\prime }|x)$ is found by maximizing the entropy

(1) $$S\left[P,Q\right]=-\int dx^{\prime }\;P\left(x^{\prime }|x\right)\log\frac{P(x^{\prime }|x)}{Q(x^{\prime }|x)}$$

relative to the joint prior $Q(x^{\prime }|x)$ subject to constraints given below. (In multidimensional integrals such as (1) the notation $dx^{\prime }$ stands for $d^{3N}x^{\prime }$.)

**The prior**. The choice of prior $Q(x^{\prime }|x)$ must reflect the state of knowledge that is common to all short steps. (It is through the constraints that the information that is specific to any particular short step will be supplied.) We adopt a prior that carries the information that the particles take infinitesimally short steps and reflects the translational and rotational invariance of the Euclidean space **X** but is otherwise uninformative. In particular, the prior expresses total ignorance about any correlations. Such a prior can itself be derived from the principle of maximum entropy. Indeed, maximize

(2) $$S\left[Q\right]=-\int dx^{\prime }\;Q\left(x^{\prime }|x\right)\log\frac{Q(x^{\prime }|x)}{\mu (x^{\prime })},$$

relative to the uniform measure $\mu (x^{\prime })$ [83], subject to normalization, and subject to the *N* independent constraints

(3) $$\langle \delta_{ab}\Delta x_{n}^{a}\Delta x_{n}^{b}\rangle =\kappa_{n},\;\left(n=1\ldots N\right),$$

where $\kappa_{n}$ are small constants and $\Delta x_{n}^{a}={x^{\prime }}_{n}^{a}-x_{n}^{a}$. The result is a product of Gaussians,

(4) $$Q\left(x^{\prime }|x\right)\propto \exp-\frac{1}{2}\sum_{n}\alpha_{n}\delta_{ab}\Delta x_{n}^{a}\Delta x_{n}^{b}\;,$$

where, to reflect translational invariance and possibly non-identical particles, the Lagrange multipliers $\alpha_{n}$ are independent of *x* but may depend on the index *n*. Eventually we will let $\alpha_{n}\to \infty$ to implement infinitesimally short steps. Next we specify the constraints that are specific to each particular short step.

**The drift potential constraint**. In Newtonian dynamics one does not need to explain why a particle perseveres in its motion in a straight line; what demands an explanation—that is, a force—is why the particle deviates from inertial motion. In ED one does not require an explanation for why the particles move; what requires an explanation is how the motion can be both directional and highly correlated. This physical information is introduced through one constraint that acts simultaneously on all particles. The constraint involves a function $\phi \left(x\right)=\phi \left(x_{1}\ldots x_{N}\right)$ on configuration space $X_{N}$ that we call the “drift” potential. We impose that the displacements $\Delta x_{n}^{a}$ are such that the expected change of the drift potential $\langle \Delta \phi \rangle$ is constrained to be

(5) $$\langle \Delta \phi \rangle =\sum_{n=1}^{N}\langle \Delta x_{n}^{a}\rangle \frac{\partial \phi }{\partial x_{n}^{a}}=\kappa^{\prime }\left(x\right),$$

where $\kappa^{\prime }\left(x\right)$ is another small but for now unspecified function. As we shall later see this information is already sufficient to construct an interesting ED. However, to reproduce the particular dynamics that describes quantum systems we must further require that the potential ϕ(x) be a multi-valued function with the topological properties of an angle—ϕ and ϕ+2π represent the same angle [84].

The physical origin of the drift potential ϕ(x) is at this point unknown so how can one justify its introduction? The idea is that identifying the relevant constraints can represent significant progress even when their physical origin remains unexplained. Indeed, with the *single* assumption of a constraint involving a drift potential we will explain and coordinate *several* features of quantum mechanics such as entanglement, the existence of complex and symplectic structures, the actual form of the Hamiltonian, and the linearity of the Schrödinger equation.

**The gauge constraints**. The single constraint (5) already leads to a rich entropic dynamics but by imposing additional constraints we can construct even more realistic models. To incorporate the effect of an external electromagnetic field we impose that for each particle *n* the expected displacement $\langle \Delta x_{n}^{a}\rangle$ will satisfy

(6) $$\langle \Delta x_{n}^{a}\rangle A_{a}\left(x_{n}\right)=\kappa_{n}^{\prime \prime }\;\mathrm{for}\;n=1\ldots N,$$

where the electromagnetic vector potential $A_{a}\left(x_{n}\right)$ is a field that lives in the 3-dimensional physical space ($x_{n}\in X$). The strength of the coupling is given by the values of the $\kappa_{n}^{\prime \prime }$. These quantities could be specified directly but, as is often the case in entropic inference, it is much more convenient to specify them indirectly in terms of the corresponding Lagrange multipliers.

**The transition probability**. An important feature of the ED model can already be discerned. The central object of the discussion so far, the transition probability $P(x^{\prime }|x)$, codifies information supplied through the prior and the constraints which makes no reference to anything earlier than the initial position *x*. Therefore ED must take the form of a Markov process.

The distribution $P(x^{\prime }|x)$ that maximizes the entropy S[P,Q] in (1) relative to (4) and subject to (5), and (6), and normalization is

(7) $$P\left(x^{\prime }|x\right)=\frac{1}{Z}\exp-\sum_{n}\left(\frac{\alpha_{n}}{2}\delta_{ab}\Delta x_{n}^{a}\Delta x_{n}^{b}-\alpha^{\prime }\left[\partial_{na}\phi -\beta_{n}A_{a}\left(x_{n}\right)\right]\Delta x_{n}^{a}\right)$$

where $\alpha^{\prime }$ and $\beta_{n}$ are Lagrange multipliers. This is conveniently written as

(8) $$P\left(x^{\prime }|x\right)=\frac{1}{Z}\exp-\sum_{n}\frac{\alpha_{n}}{2}\delta_{ab}\left(\Delta x_{n}^{a}-\Delta {\bar{x}}_{n}^{a}\right)\left(\Delta x_{n}^{b}-\Delta {\bar{x}}_{n}^{b}\right),$$

with a suitably modified normalization and

(9) $$\Delta {\bar{x}}_{n}^{a}=\frac{\alpha^{\prime }}{\alpha_{n}}\left[\partial_{na}\phi -\beta_{n}A_{a}\left(x_{n}\right)\right]=\langle \Delta x_{n}^{a}\rangle .$$

A generic displacement is expressed as a drift plus a fluctuation,

(10) $$\Delta x_{n}^{a}=\langle \Delta x_{n}^{a}\rangle +\Delta w_{n}^{a},$$

where

(11) $$\langle \Delta w_{n}^{a}\rangle =0,\;\mathrm{and}\;\langle \Delta w_{n}^{a}\Delta w_{n^{\prime }}^{b}\rangle =\frac{1}{\alpha_{n}}\delta_{nn^{\prime }}\delta^{ab},$$

The fact that the constraints (5) and (6) are not independent—both involve the same displacements $\langle \Delta x_{n}^{a}\rangle$—has turned out to be significant. We can already see in (7) and (9) that it leads to a gauge symmetry. As we shall later see the vector potential $A_{a}$ will be interpreted as the corresponding gauge connection field and the multipliers $\beta_{n}$ will be related to the electric charges through $\beta_{n}=q_{n}/\hbar c$.

## 3. Entropic Time

The task of iterating the short steps described by the transition probability (8) to predict motion over finite distances leads us to introduce a book-keeping parameter *t*, to be called time, in order to keep track of the accumulation of short steps. The construction of time involves three ingredients: (a) we must specify what we mean by an ‘instant’; (b) these instants must be ordered; and finally; (c) one must specify the interval Δt between successive instants—one must define ‘duration’.

Since the foundation for any theory of time is the theory of change, i.e., the dynamics, the notion of time constructed below will reflect the inferential nature of entropic dynamics. Such a construction we will call “entropic” time [10]. Later we will return to the question of whether and how this “entropic” time is related to the “physical” time that is measured by clocks.

### 3.1. Time as an Ordered Sequence of Instants

ED consists of a succession of short steps. Consider, for example, the *i*th step which takes the system from $x=x_{i-1}$ to $x^{\prime }=x_{i}$. Integrating the joint probability, $P(x_{i},x_{i-1})$, over $x_{i-1}$ gives

(12) $$P\left(x_{i}\right)=\int dx_{i-1}P\left(x_{i},x_{i-1}\right)=\int dx_{i-1}P\left(x_{i}|x_{i-1}\right)P\left(x_{i-1}\right).$$

No physical assumptions were involved in deriving this equation; it follows directly from the laws of probability. To establish the connection to time and dynamics we will make the physical assumption that if $P(x_{i-1})$ is interpreted as the probability of different values of $x_{i-1}$ at one “instant” labelled *t*, then we will interpret $P(x_{i})$ as the probability of values of $x_{i}$ at the next “instant” labelled $t^{\prime }$. More explicitly, if we write $P\left(x_{i-1}\right)=\rho_{t}\left(x\right)$ and $P\left(x_{i}\right)=\rho_{t^{\prime }}\left(x^{\prime }\right)$ then we have

(13) $$\rho_{t^{\prime }}\left(x^{\prime }\right)=\int dx\;P\left(x^{\prime }|x\right)\rho_{t}\left(x\right).$$

This equation defines the notion of “instant”: if the distribution $\rho_{t}\left(x\right)$ refers to one instant *t*, then the distribution $\rho_{t^{\prime }}\left(x^{\prime }\right)$ generated by $P(x^{\prime }|x)$ defines what we mean by the “next” instant $t^{\prime }$. Iterating this process defines the dynamics.

This construction of time is intimately related to information and inference. An instant is an informational state that is complete in the sense that it is specified by the information—codified into the distributions $\rho_{t}\left(x\right)$ and $P(x^{\prime }|x)$—that is sufficient for predicting the next instant. Thus, the present is defined through a sufficient amount of information such that given the present, the future is independent of the past.

In the ED framework the notions of instant and of simultaneity are intimately related to the distribution $\rho_{t}\left(x\right)$. To see how this comes about consider a single particle at the point $\vec{x}=\left(x^{1},x^{2},x^{3}\right)$. It is implicit in the notation that $x^{1}$, $x^{2}$, and $x^{3}$ occur simultaneously. When we describe a system of *N* particles by a single point $x=({\vec{x}}_{1},{\vec{x}}_{2},\ldots {\vec{x}}_{N})$ in 3N-dimensional configuration space it is also implicitly assumed that all the 3N coordinate values refer to the same instant; they are simultaneous. The very idea of a *point* in configuration space assumes simultaneity. And furthermore, whether we deal with one particle or many, a distribution such as $\rho_{t}\left(x\right)$ is meant to describe our uncertainty about the possible configurations *x* of the system at the given instant. Thus, a probability distribution $\rho_{t}\left(x\right)$ provides a criterion of simultaneity [85].

### 3.2. The Arrow of Entropic Time

The notion of time constructed according to Equation (13) is intrinsically directional. There is an absolute sense in which $\rho_{t}\left(x\right)$ is prior and $\rho_{t^{\prime }}\left(x^{\prime }\right)$ is posterior. Indeed, the same rules of probability that led us to Equation (13) can also lead us to the time-reversed evolution,

(14) $$\rho_{t}\left(x\right)=\int dx^{\prime }\;P\left(x|x^{\prime }\right)\rho_{t^{\prime }}\left(x^{\prime }\right).$$

Note, however, that there is a temporal asymmetry: while the distribution $P(x^{\prime }|x)$, Equation (7), is a Gaussian derived using the maximum entropy method, its time-reversed version $P(x|x^{\prime })$ is related to $P(x^{\prime }|x)$ by Bayes’ theorem,

(15) $$P\left(x|x^{\prime }\right)=\frac{\rho_{t}\left(x\right)}{\rho_{t^{\prime }}\left(x^{\prime }\right)}P\left(x^{\prime }|x\right),$$

which in general will not be Gaussian.

The puzzle of the arrow of time (see e.g., [86,87]) arises from the difficulty in deriving a temporal asymmetry from underlying laws of nature that are symmetric. The ED approach offers a fresh perspective on this topic because it does not assume any underlying laws of nature—whether they be symmetric or not. The asymmetry is the inevitable consequence of constructing time in a dynamics driven by entropic inference.

From the ED point of view the challenge does not consist of explaining the arrow of time—*entropic time itself only flows forward*—but rather in explaining how it comes about that despite the arrow of time some laws of physics, such as the Schrödinger equation, turn out to be time reversible. We will revisit this topic in Section 9.

### 3.3. Duration and the Sub-Quantum Motion

We have argued that the concept of time is intimately connected to the associated dynamics but at this point neither the transition probability $P(x^{\prime }|x)$ that specifies the dynamics nor the corresponding entropic time have been fully defined yet. It remains to specify how the multipliers $\alpha_{n}$ and $\alpha^{\prime }$ are related to the interval Δt between successive instants.

The basic criterion for this choice is convenience: *duration is defined so that motion looks simple*. The description of motion is simplest when it reflects the symmetry of translations in space and time. In a flat space-time this leads to an entropic time that resembles Newtonian time in that it flows “equably everywhere and everywhen.” Referring to Equations (9) and (11) we choose $\alpha^{\prime }$ and $\alpha_{n}$ to be independent of *x* and *t*, and we choose the ratio $\alpha^{\prime }/\alpha_{n}\propto \Delta t$ so that there is a well-defined drift velocity. For future convenience the proportionality constants will be expressed in terms of some particle-specific constants $m_{n}$,

(16) $$\frac{\alpha^{\prime }}{\alpha_{n}}=\frac{\hbar }{m_{n}}\Delta t,$$

where *ℏ* is an overall constant that fixes the units of the $m_{n}$s relative to the units of time. As we shall later see, the constants $m_{n}$ will eventually be identified with the particle masses while the constant *ℏ* will be identified as Planck’s constant. Having specified the ratio $\alpha^{\prime }/\alpha_{n}$ it remains to specify $\alpha_{n}$ (or $\alpha^{\prime }$). It turns out that the choice is not unique. There is a variety of motions at the sub-quantum “microscopic” level that lead to the same quantum mechanics at the “macroscopic” level.

In previous work [10,11,12] we chose $\alpha_{n}$ proportional to 1/Δt. This led to an ED in which the particles follow the highly irregular non-differentiable Brownian trajectories characteristic of an Einstein–Smoluchowski process. The first new contribution of this paper is to explore the consequences of choosing $\alpha_{n}\propto 1/\Delta t^{3}$,

(17) $$\alpha_{n}=\frac{m_{n}}{\eta \Delta t^{3}}\;,$$

where a new constant η is introduced.

It is convenient to introduce a notation tailored to configuration space. Let $x^{A}=x_{n}^{a}$, $\partial_{A}=\partial /\partial x_{n}^{a}$, and $\delta_{AB}=\delta_{nn^{\prime }}\delta_{ab}$, where the upper case indices A,B,… label both the particles $n,n^{\prime },\ldots$ and their coordinates a,b,…. Then the transition probability (8) becomes

(18) $$P\left(x^{\prime }|x\right)=\frac{1}{Z}\exp\left[-\frac{1}{2\eta \Delta t}m_{AB}\left(\frac{\Delta x^{A}}{\Delta t}-v^{A}\right)\left(\frac{\Delta x^{B}}{\Delta t}-v^{B}\right)\right],$$

where we used (9) to define the drift velocity,

(19) $$v^{A}=\frac{\langle \Delta x^{A}\rangle }{\Delta t}=m^{AB}\left[\partial_{B}\Phi -{\bar{A}}_{B}\right].$$

The drift potential is rescaled into a new variable

(20) Φ=ℏϕ

which will be called *the phase*. We also introduced the “mass” tensor and its inverse,

(21) $$m_{AB}=m_{n}\delta_{AB}=m_{n}\delta_{nn^{\prime }}\delta_{ab}\;\mathrm{and}\;m^{AB}=\frac{1}{m_{n}}\delta^{AB},$$

and ${\bar{A}}_{A}$ is a field in configuration space with components,

(22) $${\bar{A}}_{A}\left(x\right)=\hbar \beta_{n}A_{a}\left(x_{n}\right)\;,$$

A generic displacement is then written as a drift plus a fluctuation,

(23) $$\Delta x^{A}=v^{A}\Delta t+\Delta w^{A},$$

and the fluctuations $\Delta w^{A}$ are given by

(24) $$\langle \Delta w^{A}\rangle =0\;\mathrm{and}\;\langle \Delta w^{A}\Delta w^{B}\rangle =\eta m^{AB}\Delta t^{3}\;,$$

or

(25) $$\langle \left(\frac{\Delta x^{A}}{\Delta t}-v^{A}\right)\left(\frac{\Delta x^{B}}{\Delta t}-v^{B}\right)\rangle =\eta m^{AB}\Delta t.$$

It is noteworthy that $\langle \Delta x^{A}\rangle ∼O\left(\Delta t\right)$ and $\Delta w^{A}∼O\left(\Delta t^{3/2}\right)$. This means that for short steps the fluctuations are negligible and the dynamics is dominated by the drift. The particles follow trajectories that are indeterministic but differentiable. Since $\Delta w^{A}∼O\left(\Delta t^{3/2}\right)$ the limit

(26) $$V^{A}=\lim_{\Delta t\to 0}\frac{\Delta x^{A}}{\Delta t}=v^{A}$$

is well defined. In words: the actual velocities of the particles coincide with the expected or drift velocities. From Equation (19) we see that these velocities are continuous functions. The question of whether the velocities themselves are differentiable or not is trickier.

Consider two successive displacements $\Delta x=x^{\prime }-x$ followed by $\Delta x^{\prime }=x^{\prime \prime }-x^{\prime }$. The velocities are

(27) $$V^{A}=\frac{\Delta x^{A}}{\Delta t}\;\mathrm{and}\;V^{\prime }{}^{A}=\frac{\Delta x^{\prime }{}^{A}}{\Delta t}.$$

The change in velocity is given by a Langevin equation,

(28) $$\Delta V^{A}={\langle \Delta V^{A}\rangle }_{x^{\prime \prime }x^{\prime }}+\Delta U^{A},$$

where ${\langle \cdot \rangle }_{x^{\prime \prime }x^{\prime }}$ denotes taking the expectations over $x^{\prime \prime }$ using $P(x^{\prime \prime }|x^{\prime })$, and then over $x^{\prime }$ using $P(x^{\prime }|x)$, and $\Delta U^{A}$ is a fluctuation. It is straightforward to show that

(29) $${\langle \Delta V^{A}\rangle }_{x^{\prime \prime }x^{\prime }}=\left(\partial_{t}v^{A}+v^{B}\partial_{B}v^{A}\right)\;\Delta t,$$

so that the expected acceleration is given by the convective derivative of the velocity field along itself,

(30) $$\lim_{\Delta t\to 0}\frac{\langle \Delta V^{A}\rangle }{\Delta t}=\left(\partial_{t}+v^{B}\partial_{B}\right)v^{A}\;.$$

One can also show that

(31) $${\langle \Delta U^{A}\rangle }_{x^{\prime \prime }x^{\prime }}=0,\;\mathrm{and}\;{\langle \Delta U^{A}\Delta U^{B}\rangle }_{x^{\prime \prime }x^{\prime }}=2\eta m^{AB}\Delta t,$$

which means that ΔU is a Wiener process and we deal with a Brownian motion of the Oernstein–Uhlenbeck type.

We conclude this section with some general remarks.

**On the nature of clocks**. In Newtonian mechanics time is defined to simplify the dynamics. The prototype of a clock is a free particle which moves equal distances in equal times. In ED time is also defined to simplify the dynamics of free particles (for sufficiently short times all particles are free) and the prototype of a clock is a free particle too: as we see in (23) *the particle’s mean displacement increases by equal amounts in equal times*.

**On the nature of mass**. In standard quantum mechanics, “what is mass?” and “why quantum fluctuations?” are two independent mysteries. In ED the mystery is somewhat alleviated: as we see in Equation (25) mass and fluctuations are two sides of the same coin. *Mass is an inverse measure of the velocity fluctuations*.

**The information metric of configuration space**. In addition to defining the dynamics the transition probability Equation (18) serves to define the geometry of the *N*-particle configuration space, $X_{N}$. Since the physical single particle space X is described by the Euclidean metric $\delta_{ab}$ we can expect that the *N*-particle configuration space, $X_{N}=X\times \ldots \times X$, will also be flat, but for non-identical particles a question might be raised about the relative scales or weights associated with each X factor. Information geometry provides the answer.

The fact that to each point $x\in X_{N}$ there corresponds a probability distribution $P(x^{\prime }|x)$ means that to the space $X_{N}$ we can associate a statistical manifold the geometry of which (up to an overall scale factor) is uniquely determined by the information metric [17,65],

(32) $$\gamma_{AB}=\int dx^{\prime }\;P\left(x^{\prime }|x\right)\frac{\partial \log P(x^{\prime }|x)}{\partial x^{A}}\frac{\partial \log P(x^{\prime }|x)}{\partial x^{B}}.$$

Substituting Equations (18) into (32) yields

(33) $$\gamma_{AB}=\frac{1}{\eta \Delta t^{3}}m_{AB}.$$

The divergence as Δt→0 arises because the information metric measures statistical distinguishability. As Δt→0 the distributions $P(x^{\prime }|x)$ and $P(x^{\prime }|x+\Delta x)$ become more sharply peaked and increasingly easier to distinguish so that $\gamma_{AB}\to \infty$. Thus, up to a scale factor the metric of configuration space is basically the mass tensor.

The practice of describing a many-particle system as a single point in an abstract configuration space goes back to the work of H. Hertz in 1894 [88]. Historically the choice of the mass tensor as the metric of configuration space has been regarded as being convenient but of no particular significance. We can now see that the choice is not just a merely useful convention: up to an overall scale the metric follows uniquely from information geometry. Furthermore, it suggests the intriguing possibility of a deeper connection between kinetic energy and information geometry.

**Invariance under gauge transformations**. The fact that constraints (5) and (6) are not independent—they are both linear in the same displacements $\langle \Delta x_{n}^{a}\rangle$—leads to a gauge symmetry. This is evident in Equation (7) where ϕ and $A_{a}$ appear in the combination $\partial_{na}\phi -\beta_{n}A_{a}$ which is invariant under the gauge transformations,

(34) $$\begin{array}{ccc} A_{a}\left(x_{n}\right) & \to & A_{a}^{\prime }\left(x_{n}\right)=A_{a}\left(x_{n}\right)+\partial_{a}\chi \left(x_{n}\right), \end{array}$$

(35) $$\begin{array}{ccc} \phi (x) & \to & \phi^{\prime }\left(x\right)=\phi \left(x\right)+\sum_{n}\beta_{n}\chi \left(x_{n}\right). \end{array}$$

These transformations are local in 3d-space. Introducing

(36) $$\bar{\chi }\left(x\right)=\sum_{n}\hbar \beta_{n}\chi \left(x_{n}\right)\;,$$

they can be written in the *N*-particle configuration space,

(37) $$\begin{array}{ccc} {\bar{A}}_{A}\left(x\right) & \to & {\bar{A}}_{A}^{\prime }\left(x\right)={\bar{A}}_{A}\left(x\right)+\partial_{A}\bar{\chi }\left(x\right), \end{array}$$

(38) $$\begin{array}{ccc} \Phi (x) & \to & \Phi^{\prime }\left(x\right)=\Phi \left(x\right)+\bar{\chi }\left(x\right). \end{array}$$

**Interpretation:** The drift potential $\phi \left(x\right)=\phi \left({\vec{x}}_{1},{\vec{x}}_{2},\ldots \right)$ is assumed to be an “angle”–ϕ(x) and ϕ(x)+2π are meant to describe the same angle. The angle at ${\vec{x}}_{1}$ depends on the values of all the other positions ${\vec{x}}_{2},{\vec{x}}_{3},\ldots$, and the angle at ${\vec{x}}_{2}$ depends on the values of all the other positions ${\vec{x}}_{1},{\vec{x}}_{3},\ldots$, and so on. The fact that the origins from which these angles are measured can be redefined by different amounts at different places gives rise to a local gauge symmetry. To compare angles at different locations one introduces a *connection* field, the vector potential $A_{a}\left(\vec{x}\right)$. It defines which origin at $\vec{x}+\Delta \vec{x}$ is the “same” as the origin at $\vec{x}$. This is implemented by imposing that as we change origins and Φ(x) changes to $\Phi +\bar{\chi }$ then the connection transforms as $A_{a}\to A_{a}+\partial_{a}\chi$ so that the quantity $\partial_{A}\Phi -{\bar{A}}_{A}$ remains invariant.

**A fractional Brownian motion**? The choices $\alpha_{n}\propto 1/\Delta t$ and $\alpha_{n}\propto 1/\Delta t^{3}$ lead to Einstein–Smoluchowski and Oernstein–Uhlenbeck processes, respectively. For definiteness throughout the rest of this paper we will assume that the sub-quantum motion is an OU process but more general fractional Brownian motions [89] are possible. Consider

(39) $$\alpha_{n}=\frac{m_{n}}{\eta \Delta t^{\gamma }}\;,$$

where γ is some positive parameter. The corresponding transition probability (8),

(40) $$P\left(x^{\prime }|x\right)=\frac{1}{Z}\exp\left[-\frac{1}{2\eta \Delta t^{\gamma }}m_{AB}\left(\Delta x^{A}-v^{A}\Delta t\right)\left(\Delta x^{B}-v^{B}\Delta t\right)\right],$$

leads to fluctuations such that

(41) $$\langle \Delta w^{A}\rangle =0\;\mathrm{and}\;\langle \Delta w^{A}\Delta w^{B}\rangle =\eta m^{AB}\Delta t^{\gamma },$$

or

(42) $$\langle \left(\frac{\Delta x^{A}}{\Delta t}-v^{A}\right)\left(\frac{\Delta x^{B}}{\Delta t}-v^{B}\right)\rangle =\eta m^{AB}\Delta t^{\gamma -2}.$$

We will not pursue this topic further except to note that since $\langle \Delta x^{A}\rangle ∼O\left(\Delta t\right)$ and $\Delta w^{A}∼O\left(\Delta t^{\gamma /2}\right)$ for γ<2 the sub-quantum motion is dominated by fluctuations and the trajectories are non-differentiable, while for γ>2 the drift dominates and velocities are well defined.

## 4. The Evolution Equation in Differential Form

Entropic dynamics is generated by iterating Equation (13): given the information that defines one instant, the integral Equation (13) is used to construct the next instant. As so often in physics it is more convenient to rewrite the equation of evolution in differential form. The result is

(43) $$\partial_{t}\rho =-\partial_{A}\left(v^{A}\rho \right)\;,$$

where $v^{A}$ is given by (19). Before we proceed to its derivation we note that Equation (43) is a consequence of the fact that the particles follow continuous paths. Accordingly, we will follow standard practice and call it *the continuity equation*. Also note that in the OU process considered here (γ=3) the current velocity—the velocity with which the probability flows in configuration space— coincides with the drift velocity (19) and with the actual velocities of the particles (26) [90].

Next we derive (43) using a technique that is well known in diffusion theory [91]. (For an alternative derivation see [92].) The result of building up a *finite* change from an initial time $t_{0}$ to a later time *t* leads to the distribution

(44) $$\rho \left(x,t\right)=\int dx_{0}\;P\left(x,t|x_{0},t_{0}\right)\rho \left(x_{0},t_{0}\right),$$

where the finite-time transition probability, $P(x,t|x_{0},t_{0})$, is constructed by iterating the infinitesimal changes described in Equation (13),

(45) $$P\left(x,t+\Delta t|x_{0},t_{0}\right)=\int dz\;P\left(x,t+\Delta t|z,t\right)P\left(z,t|x_{0},t_{0}\right).$$

For small times Δt the distribution P(x,t+Δt|z,t), given in Equation (18), is very sharply peaked at x=z. In fact, as Δt→0 we have P(x,t+Δt|z,t)→δ(x−z). Such singular behavior cannot be handled directly by Taylor expanding in *z* about the point *x*. Instead one follows an indirect procedure. Multiply by a smooth test function f(x) and integrate over *x*,

(46) $$\int dx\;P\left(x,t+\Delta t|x_{0},t_{0}\right)f\left(x\right)=\int dz\left[\int dx\;P(x,t+\Delta t|z,t)f(x)\right]P\left(z,t|x_{0},t_{0}\right).$$

The test function f(x) is assumed sufficiently smooth precisely so that it can be expanded about *z*. Then as Δt→0 the integral in the brackets, dropping all terms of order higher than Δt, is

(47) $$\begin{array}{cc} \left[\cdots \right] & =\int dx\;P\left(x,t+\Delta t|z,t\right)\left(f\left(z\right)+\frac{\partial f}{\partial z^{A}}\left(x^{A}-z^{A}\right)+\ldots \right)\\ \; & =f\left(z\right)+v^{A}\left(z\right)\Delta t\frac{\partial f}{\partial z^{A}}+\ldots \end{array}$$

where we used Equation (23). Next substitute (47) into the right hand side of (46), divide by Δt, and let Δt→0. Since f(x) is arbitrary the result is

(48) $$\partial_{t}P\left(x,t|x_{0},t_{0}\right)=-\partial_{A}\left[v^{A}\left(x\right)P\left(x,t|x_{0},t_{0}\right)\right],$$

which is the continuity equation for the finite-time transition probability. Differentiating Equation (44) with respect to *t*, and substituting (48) completes the derivation of the continuity Equation (43).

The continuity Equation (43) can be written in another equivalent but very suggestive form involving functional derivatives. For some suitably chosen functional $\tilde{H}\left[\rho ,\Phi \right]$ we have

(49) $$\partial_{t}\rho \left(x\right)=-\partial_{A}\left[\rho m^{AB}\left(\partial_{B}\Phi -{\bar{A}}_{B}\right)\right]=\frac{\delta \tilde{H}}{\delta \Phi (x)}\;.$$

It is easy to check that the appropriate functional $\tilde{H}$ is

(50) $$\tilde{H}\left[\rho ,\Phi \right]=\int dx\;\frac{1}{2}\rho m^{AB}\left(\partial_{A}\Phi -{\bar{A}}_{A}\right)\left(\partial_{B}\Phi -{\bar{A}}_{B}\right)+F\left[\rho \right],$$

where the unspecified functional F[ρ] is an integration constant [93].

The continuity Equation (49) describes a somewhat peculiar OU Brownian motion in which the probability density ρ(x) is driven by the non-dynamical fields Φ, and $\bar{A}$. This is an interesting ED in its own right but it is not QM. Indeed, a *quantum* dynamics consists in the coupled evolution of two dynamical fields: the density ρ(x) and the phase of the wave function. This second field can be naturally introduced into ED by allowing the phase field Φ in (19) to become dynamical which amounts to an ED in which the constraint (5) is continuously updated at each instant in time. Our next topic is to propose the appropriate updating criterion. It yields an ED in which the phase field Φ guides the evolution of ρ, and in return, the evolving ρ reacts back and induces a change in Φ.

## 5. The Epistemic Phase Space

In ED we deal with two configuration spaces. One is the *ontic configuration space* $X_{N}=X\times X\times \ldots$ of all particle positions, $x=\left(x_{1}\ldots x_{N}\right)\in X_{N}$. The other is the *epistemic configuration space* or *e-configuration space* **P** of all normalized probabilities,

(51) $$P=\left\{\rho \left|\rho (x)\geq 0;\int dx\rho (x)=1\right.\right\}.$$

To formulate the coupled dynamics of ρ and Φ we need a framework to study paths in the larger space {ρ,Φ} that we will call the *epistemic phase space* or *e-phase space*.

Given any manifold such as **P** the associated tangent and cotangent bundles, respectively TP and $T^{*}P$, are geometric objects that are always available to us independently of any physical considerations. Both are manifolds in their own right but the cotangent bundle $T^{*}P$—the space of all probabilities and all covectors—is of particular interest because it comes automatically endowed with a rich geometrical structure [56,57,58,59,60,61,62]. The point is that cotangent bundles are symplectic manifolds and this singles out as “natural” those dynamical laws that happen to preserve some privileged symplectic form. This observation will lead us to identify e-phase space {ρ,Φ} with the cotangent bundle $T^{*}P$ and provides the natural criterion for updating constraints, that is, for updating the phase Φ [94].

### 5.1. Notation: Vectors, Covectors, Etc.

A point $X\in T^{*}P$ will be represented as

(52) $$X=\left(\rho (x),\pi (x)\right)=\left(\rho^{x},\pi_{x}\right),$$

where $\rho^{x}$ represents coordinates on the base manifold P and $\pi_{x}$ represents some generic coordinates on the space $T^{*}P_{\rho }$ that is cotangent to **P** at the point ρ. Curves in $T^{*}P$ allow us to define vectors. Let X=X(λ) be a curve parametrized by λ, then the vector $\bar{V}$ tangent to the curve at X=(ρ,π) has components $d\rho^{x}/d\lambda$ and $d\pi_{x}/d\lambda$, and is written

(53) $$\bar{V}=\frac{d}{d\lambda }=\int dx\;\left[\frac{d\rho^{x}}{d\lambda }\frac{\delta }{\delta \rho^{x}}+\frac{d\pi_{x}}{d\lambda }\frac{\delta }{\delta \pi_{x}}\right],$$

where $\delta /\delta \rho^{x}$ and $\delta /\delta \pi_{x}$ are the basis vectors. The directional derivative of a functional F[X] along the curve X(λ) is

(54) $$\frac{dF}{d\lambda }=\tilde{\nabla }F\left[\bar{V}\right]=\int dx\;\left[\frac{\delta F}{\delta \rho^{x}}\frac{d\rho^{x}}{d\lambda }+\frac{\delta F}{\delta \pi_{x}}\frac{d\pi_{x}}{d\lambda }\right],$$

where $\tilde{\nabla }$ is the functional gradient in $T^{*}P$, i.e., the gradient of a generic functional F[X]=F[ρ,π] is

(55) $$\tilde{\nabla }F=\int dx\;\left[\frac{\delta F}{\delta \rho^{x}}\tilde{\nabla }\rho^{x}+\frac{\delta F}{\delta \pi_{x}}\tilde{\nabla }\pi_{x}\right].$$

The tilde ‘˜’ serves to distinguish the functional gradient $\tilde{\nabla }$ from the spatial gradient $\nabla f=\partial_{a}f\nabla x^{a}$ on $R^{3}$.

The fact that the space **P** is constrained to normalized probabilities means that the coordinates $\rho^{x}$ are not independent. This technical difficulty is handled by embedding the ∞-dimensional manifold P in a (∞ + 1)-dimensional manifold $P^{+1}$ where the coordinates $\rho^{x}$ are unconstrained [95]. Thus, strictly, $\tilde{\nabla }F$ is a covector on $T^{*}P^{+1}$, i.e., $\tilde{\nabla }F\in T^{*}{\left(T^{*}P^{+1}\right)}_{X}$ and $\tilde{\nabla }\rho^{x}$ and $\tilde{\nabla }\pi_{x}$ are the corresponding basis covectors. Nevertheless, the gradient $\tilde{\nabla }F$ will yield the desired directional derivatives (54) on $T^{*}P$ provided its action is restricted to vectors $\bar{V}$ that are tangent to the manifold P. Such tangent vectors are constrained to obey

(56) $$\frac{d}{d\lambda }\int dx\rho \left(x\right)=\int dx\;\frac{d\rho^{x}}{d\lambda }=0.$$

Instead of keeping separate track of the $\rho^{x}$ and $\pi_{x}$ coordinates it is more convenient to combine them into a single index. A point X=(ρ,π) will then be labelled by its coordinates

(57) $$X^{I}=\left(X^{1x},X^{2x}\right)=\left(\rho^{x},\pi_{x}\right).$$

We will use capital letters from the middle of the Latin alphabet (I,J,K…); I=(α,x) is a composite index where α=1,2 keeps track of whether *x* is an upper index (α=1) or a lower index (α=2) [96]. Then Equations (53)–(55) are written as

(58) $$\bar{V}=V^{I}\frac{\delta }{\delta X^{I}},\;\mathrm{where}\;V^{I}=\frac{dX^{I}}{d\lambda }=\left[\begin{array}{c} d\rho^{x}/d\lambda \\ d\pi_{x}/d\lambda \end{array}\right],$$

(59) $$\frac{dF}{d\lambda }=\tilde{\nabla }F\left[\bar{V}\right]=\frac{\delta F}{\delta X^{I}}V^{I}\;\mathrm{and}\;\tilde{\nabla }F=\frac{\delta F}{\delta X^{I}}\tilde{\nabla }X^{I},$$

where the repeated indices indicate a summation over α and an integration over *x*.

### 5.2. The Symplectic Form in ED

In classical mechanics with configuration space $\left\{q^{i}\right\}$ the Lagrangian $L(q,\dot{q})$ is a function on the tangent bundle while the Hamiltonian H(q,p) is a function on the cotangent bundle [97,98]. A symplectic form provides a mapping from the tangent to the cotangent bundles. Given a Lagrangian the map is defined by $p_{i}=\partial L/\partial {\dot{q}}^{i}$ and this automatically defines the corresponding symplectic form. In ED there is no Lagrangian so to define the symplectic map we must look elsewhere. We propose that the role played by the Lagrangian in classical mechanics will in ED be played by the continuity Equation (49).

The fact that the preservation of a symplectic structure must reproduce the continuity equation leads us to identify the phase $\Phi_{x}$ as the momentum canonically conjugate to $\rho^{x}$. This identification of the e-phase space {ρ,Φ} with $T^{*}P$ is highly non-trivial. It amounts to asserting that the phase $\Phi_{x}$ transforms as the components of a Poincare 1-form

(60) $$\theta =\int dx\;\Phi_{x}d\rho^{x},$$

where d is the exterior derivative and the corresponding symplectic 2-form Ω=−dθ is

(61) $$\Omega =\int dx\;d\rho^{x}\wedge d\Phi_{x}=\int dx\;\left[\tilde{\nabla }\rho^{x}⊗\tilde{\nabla }\Phi_{x}-\tilde{\nabla }\Phi_{x}⊗\tilde{\nabla }\rho^{x}\right].$$

By construction Ω is exact (Ω=−dθ) and closed (dΩ=0). The action of Ω[·,·] on two vectors $\bar{V}=d/d\lambda$ and $\bar{U}=d/d\mu$ is given by

(62) $$\Omega \left[\bar{V},\bar{U}\right]=\int dx\;\left[V^{1x}U^{2x}-V^{2x}U^{1x}\right]=\Omega_{IJ}V^{I}U^{J},$$

so that the components of Ω are

(63) $$\Omega_{IJ}=\Omega_{\alpha x,\beta x^{\prime }}=\left[\begin{array}{cc} 0 & 1\\ -1 & 0 \end{array}\right]\delta \left(x,x^{\prime }\right).$$

### 5.3. Hamiltonian Flows and Poisson Brackets

Next we reproduce the ∞-dimensional $T^{*}P$ analogues of results that are standard in finite-dimensional classical mechanics [97,98]. Given a vector field $\bar{V}\left[X\right]$ in e-phase space we can integrate $V^{I}\left[X\right]=dX^{I}/d\lambda$ to find its integral curves $X^{I}=X^{I}\left(\lambda \right)$. We are particularly interested in those vector fields that generate flows that preserve the symplectic structure,

(64) $${£}_{V}\Omega =0,$$

where the Lie derivative is given by

(65) $${\left({£}_{V}\Omega \right)}_{IJ}=V^{K}{\tilde{\nabla }}_{K}\Omega_{IJ}+\Omega_{KJ}{\tilde{\nabla }}_{I}V^{K}+\Omega_{IK}{\tilde{\nabla }}_{J}V^{K}\;.$$

Since by Equation (63) the components $\Omega_{IJ}$ are constant, ${\tilde{\nabla }}_{K}\Omega_{IJ}=0$, we can rewrite ${£}_{V}\Omega$ as

(66) $${\left({£}_{V}\Omega \right)}_{IJ}={\tilde{\nabla }}_{I}\left(\Omega_{KJ}V^{K}\right)-{\tilde{\nabla }}_{J}\left(\Omega_{KI}V^{K}\right),$$

which is the exterior derivative (basically, the curl) of the covector $\Omega_{KI}V^{K}$. By Poincare’s lemma, requiring ${£}_{V}\Omega =0$ (a vanishing curl) implies that $\Omega_{KI}V^{K}$ is the gradient of a scalar function, which we will denote $\tilde{V}\left[X\right]$,

(67) $$\Omega_{KI}V^{K}={\tilde{\nabla }}_{I}\tilde{V}.$$

Using (63) this is more explicitly written as

(68) $$\int dx\;\left[\frac{d\rho^{x}}{d\lambda }\tilde{\nabla }\Phi_{x}-\frac{d\Phi_{x}}{d\lambda }\tilde{\nabla }\rho^{x}\right]=\int dx\;\left[\frac{\delta \tilde{V}}{\delta \rho^{x}}\tilde{\nabla }\rho^{x}+\frac{\delta \tilde{V}}{\delta \Phi_{x}}\tilde{\nabla }\Phi_{x}\right],$$

or

(69) $$\frac{d\rho^{x}}{d\lambda }=\frac{\delta \tilde{V}}{\delta \Phi_{x}}\;\mathrm{and}\;\frac{d\Phi_{x}}{d\lambda }=-\frac{\delta \tilde{V}}{\delta \rho^{x}},$$

which we recognize as Hamilton’s equations for a Hamiltonian function $\tilde{V}$. This justifies calling $\bar{V}$ the Hamiltonian vector field associated with the Hamiltonian function $\tilde{V}$.

From (62), the action of the symplectic form Ω on two Hamiltonian vector fields $\bar{V}=d/d\lambda$ and $\bar{U}=d/d\mu$ generated respectively by $\tilde{V}$ and $\tilde{U}$ is

(70) $$\Omega \left[\bar{V},\bar{U}\right]=\int dx\;\left[\frac{d\rho^{x}}{d\lambda }\frac{d\Phi_{x}}{d\mu }-\frac{d\Phi_{x}}{d\lambda }\frac{d\rho^{x}}{d\mu }\right]\;,$$

which, using (69), gives

(71) $$\Omega \left[\bar{V},\bar{U}\right]=\int dx\;\left[\frac{\delta \tilde{V}}{\delta \rho^{x}}\frac{\delta \tilde{U}}{\delta \Phi_{x}}-\frac{\delta \tilde{V}}{\delta \Phi_{x}}\frac{\delta \tilde{U}}{\delta \rho^{x}}\right]\overset{\mathrm{def}}{=}\left\{\tilde{V},\tilde{U}\right\},$$

where on the right we introduced the Poisson bracket notation.

To summarize these results: (1) The condition for a flow generated by the vector field $V^{I}$ to preserve the symplectic structure, ${£}_{V}\Omega =0$, is that $V^{I}$ be the Hamiltonian vector field associated to a Hamiltonian function $\tilde{V}$, Equation (69),

(72) $$V^{I}=\frac{dX^{I}}{d\lambda }=\left\{X^{I},\tilde{V}\right\}.$$

(2) The action of Ω on two Hamiltonian vector fields (71) is the Poisson bracket of the associated Hamiltonian functions,

(73) $$\Omega \left[\bar{V},\bar{U}\right]=\Omega_{IJ}V^{I}U^{J}=\left\{\tilde{V},\tilde{U}\right\}.$$

We conclude that the ED that preserves the symplectic structure Ω and reproduces the continuity Equation (49) is described by the Hamiltonian flow of the scalar functional $\tilde{H}$ in (50). However, the full dynamics, which will obey the Hamiltonian evolution equations

(74) $$\partial_{t}\rho^{x}=\frac{\delta \tilde{H}}{\delta \Phi_{x}}\;\mathrm{and}\;\partial_{t}\Phi_{x}=-\frac{\delta \tilde{H}}{\delta \rho^{x}},$$

is not yet fully determined because the integration constant F[ρ] in (50) remains to be specified.

### 5.4. The Normalization Constraint

Since the particular flow that we will associate with time evolution is required to reproduce the continuity equation it will also preserve the normalization constraint,

(75) $$\tilde{N}=0\;\mathrm{where}\;\tilde{N}=1-\left|\rho \right|\;\mathrm{and}\;\left|\rho \right|\overset{\mathrm{def}}{=}\int dx\;\rho \left(x\right).$$

Indeed, one can check that

(76) $$\partial_{t}\tilde{N}=\left\{\tilde{N},\tilde{H}\right\}=0.$$

The Hamiltonian flow (72) generated by $\tilde{N}$ and parametrized by α is given by the vector field

(77) $$\bar{N}=N^{I}\frac{\delta }{\delta X^{I}}\;\mathrm{with}\;N^{I}=\frac{dX^{I}}{d\alpha }=\left\{X^{I},\tilde{N}\right\},$$

or, more explicitly,

(78) $$N^{1x}=\frac{d\rho^{x}}{d\alpha }=0\;\mathrm{and}\;N^{2x}=\frac{d\Phi_{x}}{d\alpha }=1.$$

The conservation of $\tilde{N}$, Equation (76), implies that $\tilde{N}$ is the generator of a symmetry, namely

(79) $$\frac{d\tilde{H}}{d\alpha }=\left\{\tilde{H},\tilde{N}\right\}=0.$$

Integrating (78) one finds the integral curves generated by $\tilde{N}$,

(80) $$\rho^{x}\left(\alpha \right)=\rho^{x}\left(0\right)\;\mathrm{and}\;\Phi_{x}\left(\alpha \right)=\Phi_{x}\left(0\right)+\alpha \;.$$

This shows that the symmetry generated by $\tilde{N}$ is to shift the phase Φ by a constant α without otherwise changing the dynamics. This was, of course, already evident in the continuity Equation (43) with (19) but the implications are very significant. Not only does the constraint $\tilde{N}=0$ reduce by one the (infinite) number of independent $\rho^{x}$ degrees of freedom but the actual number of $\Phi_{x}$s is also reduced by one because for any value of α the phases $\Phi_{x}+\alpha$ and $\Phi_{x}$ correspond to the same state. (This is the ED analogue of the fact that in QM states are represented by rays rather than vectors in a Hilbert space.) An immediate consequence is that two vectors $\bar{U}$ and $\bar{V}$ at *X* that differ by a vector proportional to $\bar{N}$,

(81) $$\bar{U}=\bar{V}+k\bar{N},$$

are “physically” equivalent. In particular the vector $\bar{N}$ is equivalent to zero.

The phase space of interest is $T^{*}P$ but to handle the constraint |ρ|=1 we have been led to using coordinates that are more appropriate to the larger embedding space $T^{*}P^{+1}$. The price we pay for introducing one superfluous coordinate is to also introduce a superfluous momentum. We eliminate the extra coordinate by imposing the constraint $\tilde{N}=0$. We eliminate the extra momentum by declaring it unphysical. All vectors that differ by a vector along the gauge direction $\bar{N}$ are declared equivalent; they belong to the same equivalence class. The result is a global gauge symmetry.

An equivalence class can be represented by any one of its members and choosing a convenient representative amounts to fixing the gauge. As we shall see below a convenient gauge condition is to impose

(82) $$\int dx\;\rho^{x}V^{2x}=0\;\mathrm{or}\;\langle V^{2}\rangle =0,$$

so that the representative “Tangent Gauge-Fixed” vectors (which we shall refer to as TGF vectors) will satisfy two conditions, Equations (56) and (82),

(83) $$\left|V^{1}\right|=\int dx\;V^{1x}=0\;\mathrm{and}\;\langle V^{2}\rangle =\int dx\;\rho^{x}V^{2x}=0.$$

The first condition enforces a flow tangent to the $\left|\rho \right|=1$ surface; the second eliminates a superfluous vector component along the gauge direction $\bar{N}$.

We end this section with a comment on the symplectic form Ω which is non-degenerate on $T^{*}P^{+1}$ but at first sight appears to be degenerate on $T^{*}P$. Indeed, we have $\Omega (\bar{N},\bar{V})=0$ for any tangent vector $\bar{V}$. However, we must recall that $\bar{N}$ is equivalent to 0. In fact, since the TGF equivalent of $\bar{N}$ is 0, Ω is not degenerate on $T^{*}P$.

## 6. The Information Geometry of E-Phase Space

The construction of the ensemble Hamiltonian $\tilde{H}$—or *e-Hamiltonian*—is motivated as follows. The goal of dynamics is to determine the evolution of the state $\left(\rho_{t},\Phi_{t}\right)$. From a given initial state $\left(\rho_{0},\Phi_{0}\right)$ two slightly different Hamiltonians will lead to slightly different final states, say $\left(\rho_{t},\Phi_{t}\right)$ or $\left(\rho_{t}+\delta \rho_{t},\Phi_{t}+\delta \Phi_{t}\right)$. Will these small changes make any difference? Can we quantify the extent to which we can *distinguish* between two neighboring states? This is precisely the kind of question that metrics are designed to address. It is then natural that $\tilde{H}$ be in some way related to some choice of metric. But although P is naturally endowed with a unique information metric the space $T^{*}P$ has none. Thus, our next goal is to construct a metric for $T^{*}P$.

Once a metric structure is in place we can ask: does the distance between two neighboring states—the extent to which we can *distinguish* them—grow, stay the same, or diminish over time? There are many possibilities here but for pragmatic (and esthetic) reasons we are led to consider the simplest form of dynamics—one that preserves the metric. This leads us to study the Hamilton flows (those that preserve the symplectic structure) that are also Killing flows (those flows that preserve the metric structure).

In ED entropic time is constructed so that time (duration) is defined by a clock provided by the system itself. This leads to require that the generator $\tilde{H}$ of time translations be defined in terms of the very same clock that provides the measure of time. Thus, the third and final ingredient in the construction of $\tilde{H}$ is the requirement is that the e-Hamiltonian agree with (50) to reproduce the evolution of ρ given by the continuity Equation (49).

In this section, our goal is to transform e-phase space $T^{*}P$ from a manifold that is merely symplectic to a manifold that is both symplectic and Riemannian. The implementation of the other two requirements on $\tilde{H}$—that it generates a Hamilton–Killing flow and that it agrees with the ED continuity equation—will be tackled in Section 7 and Section 8.

### 6.1. The Metric on the Embedding Space $T^{*}P^{+1}$

The configuration space P is a metric space. Our goal here is to extend its metric—given by information geometry—to the full cotangent bundle, $T^{*}P$. It is convenient to first recall one derivation of the information metric. In the discrete case the statistical manifold is the *k*-simplex $\Sigma =\{p=\left(p^{0}\ldots p^{k}\right):\sum_{i=0}^{k}p^{i}=1\}$. The basic idea is to find the most general metric consistent with a certain symmetry requirement. To suggest what that symmetry might be we change to new coordinates $\xi^{i}={\left(p^{i}\right)}^{1/2}$. In these new coordinates the equation for the *k*-simplex Σ—the normalization condition—reads $\sum_{i=0}^{k}{\left(\xi^{i}\right)}^{2}=1$ which suggests the equation of a sphere.

We take this hint seriously and *declare* that the *k*-simplex is a *k*-sphere embedded in a generic (k+1)-dimensional spherically symmetric space $\Sigma^{+1}$ [99]. In the $\xi^{i}$ coordinates the metric of $\Sigma^{+1}$ is of the form

(84) $$d\ell^{2}=\left[a(|p|)-b(|p|)\right]{\left(\sum_{i=0}^{k}\xi^{i}d\xi^{i}\right)}^{2}+\left|p|b(|p|\right)\sum_{i=0}^{k}{\left(d\xi^{i}\right)}^{2},$$

where a(|p|) and b(|p|) are two arbitrary smooth and positive functions of $|p|=\sum_{i=0}^{k}p^{i}$. Expressed in terms of the original $p^{i}$ coordinates the metric of $\Sigma^{+1}$ is

(85) $$d\ell^{2}=\left[a(|p|)-b(|p|)\right]{\left(\sum_{i=0}^{k}dp^{i}\right)}^{2}+\left|p|b(|p|\right)\sum_{i=0}^{k}\frac{1}{p^{i}}{\left(dp^{i}\right)}^{2}.$$

The restriction to normalized states, |p|=1 with displacements tangent to the simplex, $\sum_{i=0}^{k}dp^{i}=0$, gives the information metric induced on the *k*-simplex Σ,

(86) $$d\ell^{2}=b\left(1\right)\sum_{i=0}^{k}\frac{1}{p^{i}}{\left(dp^{i}\right)}^{2}.$$

The overall constant b(1) is not important; it amounts to a choice of the units of distance.

To extend the information metric from the *k*-simplex Σ to its cotangent bundle $T^{*}\Sigma$ we focus on the embedding spaces $\Sigma^{+1}$ and $T^{*}\Sigma^{+1}$ and require that

(a) the metric on $T^{*}\Sigma^{+1}$ be compatible with the metric on $\Sigma^{+1}$; and
(b) that the spherical symmetry of the (k+1)-dimensional space $\Sigma^{+1}$ be enlarged to full spherical symmetry for the 2(k+1)-dimensional space $T^{*}\Sigma^{+1}$.

The simplest way to implement (a) is to follow as closely as possible the derivation that led to (85). The fact that Φ inherits from the drift potential ϕ the topological structure of an angle suggests introducing new coordinates,

(87) $$\xi^{i}={\left(p^{i}\right)}^{1/2}\cos\Phi_{i}/\hbar \;\mathrm{and}\;\;\eta^{i}={\left(p^{i}\right)}^{1/2}\sin\Phi_{i}/\hbar .$$

Then the normalization condition reads

(88) $$|p|=\sum_{i=0}^{k}\;p^{i}=\sum_{i=0}^{k}\left[{\left(\xi^{i}\right)}^{2}+{\left(\eta^{i}\right)}^{2}\right]=1$$

which suggests the equation of a (2k+1)-sphere embedded in 2(k+1) dimensions. To implement (b) we take this spherical symmetry seriously. The most general metric in the embedding space that is invariant under rotations is

(89) $$\begin{array}{ccc} d\ell^{2} & = & \left[a(|p|)-b(|p|)\right]{\left[\sum_{i=0}^{k}\left(\xi^{i}d\xi^{i}+\eta^{i}d\eta^{i}\right)\right]}^{2}\\ & & +\left|p|b(|p|\right)\sum_{i=0}^{k}\left[{\left(d\xi^{i}\right)}^{2}+{\left(d\eta^{i}\right)}^{2}\right], \end{array}$$

where the two functions a(|p|) and b(|p|) are smooth and positive but otherwise arbitrary. Therefore, changing back to the $\left(p^{i},\Phi_{i}\right)$ coordinates, the most general rotationally invariant metric for the embedding space $T^{*}\Sigma^{+1}$ is

(90) $$\begin{array}{ccc} d\ell^{2} & = & \frac{1}{4}\left[a(|p|)-b(|p|)\right]{\left[\sum_{i=0}^{k}dp^{i}\right]}^{2}\\ & & +\left|p|b(|p|\right)\frac{1}{2\hbar }\sum_{i=0}^{k}\left[\frac{\hbar }{2p^{i}}{\left(dp^{i}\right)}^{2}+\frac{2p^{i}}{\hbar }{\left(d\Phi_{i}\right)}^{2}\right]. \end{array}$$

Generalizing from the finite-dimensional case to the ∞-dimensional case yields the metric on the spherically symmetric space $T^{*}P^{+1}$,

(91) $$\delta {\tilde{\ell }}^{2}=A{\left[\int dx\;\delta \rho_{x}\right]}^{2}+B\int dx\;\left[\frac{\hbar }{2\rho_{x}}{\left(\delta \rho_{x}\right)}^{2}+\frac{2\rho_{x}}{\hbar }{\left(\delta \Phi_{x}\right)}^{2}\right].$$

where we set

(92) $$A\left(|\rho |\right)=\frac{1}{4}\left[a(|\rho |)-b(|\rho |)\right]\;\mathrm{and}\;B\left(|\rho |\right)=\frac{1}{2\hbar }|\rho |b(|\rho |).$$

### 6.2. The Metric Induced on $T^{*}P$

As we saw in Section 5.4 the normalization constraint |ρ|=1 induces a symmetry—points with phases differing by a constant are identified. Therefore, the e-phase space $T^{*}P$ can be obtained from the spherically symmetric space $T^{*}P^{+1}$ by the restriction |ρ|=1 and by identifying points $\left(\rho_{x},\Phi_{x}\right)$ and $\left(\rho_{x},\Phi_{x}+\alpha \right)$ that lie on the same gauge orbit, or on the same ray.

Consider two neighboring points $\left(\rho_{x},\Phi_{x}\right)$ and $\left(\rho_{x}^{\prime },\Phi_{x}^{\prime }\right)$. The metric induced on $T^{*}P$ is defined as the shortest $T^{*}P^{+1}$ distance between $\left(\rho_{x},\Phi_{x}\right)$ and points on the ray defined by $\left(\rho_{x}^{\prime },\Phi_{x}^{\prime }\right)$. Setting |δρ|=0 the $T^{*}P^{+1}$ distance between $\left(\rho_{x},\Phi_{x}\right)$ and $\left(\rho_{x}+\delta \rho_{x},\Phi_{x}+\delta \Phi_{x}+\delta \alpha \right)$ is given by

(93) $$\delta {\tilde{\ell }}^{2}=B\left(1\right)\int dx\;\left[\frac{\hbar }{2\rho_{x}}{\left(\delta \rho_{x}\right)}^{2}+\frac{2\rho_{x}}{\hbar }{\left(\delta \Phi_{x}+\delta \alpha \right)}^{2}\right].$$

Let

(94) $$\delta {\tilde{s}}^{2}=\min_{\delta \alpha }\delta {\tilde{\ell }}^{2}.$$

Minimizing over δα gives the metric on $T^{*}P$,

(95) $$\delta {\tilde{s}}^{2}=\int dx\;\left[\frac{\hbar }{2\rho_{x}}{\left(\delta \rho_{x}\right)}^{2}+\frac{2\rho_{x}}{\hbar }{\left(\delta \Phi_{x}-\langle \delta \Phi \rangle \right)}^{2}\right],$$

where we set B(1)=1 which amounts to a choice of units of length. This metric is known as the Fubini–Study metric.

The scalar product between two vectors $\bar{V}$ and $\bar{U}$ is

(96) $$G\left(\bar{V},\bar{U}\right)=\int dx\;\left[\frac{\hbar }{2\rho_{x}}V^{1x}U^{1x}+\frac{2\rho_{x}}{\hbar }\left(V^{2x}-\langle V^{2}\rangle \right)\left(U^{2x}-\langle U^{2}\rangle \right)\right].$$

It is at this point that we recognize the convenience of imposing the TGF gauge condition (83): the scalar product simplifies to

(97) $$G\left(\bar{V},\bar{U}\right)=\int dx\;\left[\frac{\hbar }{2\rho_{x}}V^{1x}U^{1x}+\frac{2\rho_{x}}{\hbar }V^{2x}U^{2x}\right].$$

An analogous expression can be written for the length $\delta \tilde{s}$ of a displacement $\left(\delta \rho_{x},\delta \Phi_{x}\right)$,

(98) $$\delta {\tilde{s}}^{2}=\int dx\;\left[\frac{\hbar }{2\rho_{x}}{\left(\delta \rho_{x}\right)}^{2}+\frac{2\rho_{x}}{\hbar }{\left(\delta \Phi_{x}\right)}^{2}\right],$$

where it is understood that $\left(\delta \rho_{x},\delta \Phi_{x}\right)$ satisfies the TGF condition

(99) |δρ|=0and〈δΦ〉=0.

In index notation the metric (98) of $T^{*}P$ is written as

(100) $$\delta {\tilde{s}}^{2}=G_{IJ}\delta X^{I}\delta X^{J}=\int dxdx^{\prime }G_{\alpha x,\beta x^{\prime }}\delta X^{x\alpha }\delta X^{x^{\prime }\beta }$$

where the metric tensor $G_{IJ}$ is

(101) $$G_{IJ}=G_{\alpha x,\beta x^{\prime }}=\left[\begin{array}{cc} \frac{\hbar }{2\rho_{x}}\delta_{xx^{\prime }} & 0\\ 0 & \frac{2}{\hbar }\rho_{x}\delta_{xx^{\prime }} \end{array}\right].$$

The tensor $G_{IJ}$ in Equation (101) can act on arbitrary vectors whether they satisfy the TGF condition or not. It is only when $G_{IJ}$ acts on TGF vectors that it is interpreted as a metric tensor on $T^{*}P$.

### 6.3. A Complex Structure

Next we contract the symplectic form $\Omega_{IJ}$, Equation (63), with the inverse of the metric tensor,

(102) $$G^{IJ}=G^{\alpha x,\beta x^{\prime }}=\left[\begin{array}{cc} \frac{2}{\hbar }\rho_{x}\delta_{xx^{\prime }} & 0\\ 0 & \frac{\hbar }{2\rho_{x}}\delta_{xx^{\prime }} \end{array}\right].$$

The result is a mixed tensor *J* with components

(103) $$J^{I}{}_{J}=-G^{IK}\Omega_{KJ}=\left[\begin{array}{cc} 0 & -\frac{2}{\hbar }\rho_{x}\delta_{xx^{\prime }}\\ \frac{\hbar }{2\rho_{x}}\delta_{xx^{\prime }} & 0 \end{array}\right].$$

(The reason for introducing an additional negative sign will become clear below.) The tensor $J^{I}{}_{J}$ maps vectors to vectors—as any mixed (1,1) tensor should. What makes the tensor *J* special is that—as one can easily check— its action on a TGF vector $\bar{V}$ yields another vector $J\bar{V}$ that is also TGF and, furthermore, its square is

(104) $$J^{I}{}_{K}J^{K}{}_{J}=-\delta_{xx^{\prime }}\left[\begin{array}{cc} 1 & 0\\ 0 & 1 \end{array}\right]=-\delta^{I}{}_{J}.$$

In words, when acting on vectors tangent to $T^{*}P$ the action of $J^{2}$ (or $\Omega^{2}$) is equivalent to multiplying by −1. This means that *J* plays the role of a complex structure.

We conclude that the cotangent bundle $T^{*}P$ has a symplectic structure Ω, as all cotangent bundles do; that it can be given a Riemannian structure $G_{IJ}$; and that the mixed tensor *J* provides it with a complex structure.

### 6.4. Complex Coordinates

The fact that $T^{*}P$ is endowed with a complex structure suggests introducing complex coordinates,

(105) $$\Psi_{x}=\rho_{x}^{1/2}\exp i\Phi_{x}/\hbar \;,$$

so that a point Ψ∈$T^{*}P^{+1}$ has coordinates

(106) Ψμx=Ψ1xΨ2x=ΨxiℏΨx*,

where the index μ takes two values, μ=1,2.

We can check that the transformation from real coordinates (ρ,Φ) to complex coordinates $\left(\Psi ,i\hbar \Psi^{*}\right)$ is canonical. Indeed, the action of Ω on two infinitesimal vectors $\delta X^{I}$ and $\delta^{\prime }X^{J}$ is

$$\Omega_{IJ}\delta X^{I}\delta^{\prime }X^{J}=\int dx\;\left(\delta \rho_{x}\delta^{\prime }\Phi_{x}-\delta \Phi_{x}\delta^{\prime }\rho_{x}\right)\;,$$

which, when expressed in Ψ coordinates, becomes

(107) $$\Omega_{IJ}\delta X^{I}\delta^{\prime }X^{J}=\int dx\;\left(\delta \Psi \delta^{\prime }i\hbar \Psi^{*}-\delta i\hbar \Psi^{*}\delta^{\prime }\Psi \right)=\Omega_{\mu x,\nu x^{\prime }}\delta \Psi^{\mu x}\delta \Psi^{\nu x^{\prime }}$$

where

(108) $$\Omega_{\mu x,\nu x^{\prime }}=\left[\begin{array}{cc} 0 & 1\\ -1 & 0 \end{array}\right]\delta_{xx^{\prime }},$$

retains the same form as (63).

Expressed in Ψ coordinates the Hamiltonian flow generated by the normalization constraint (75),

(109) $$\tilde{N}=0\;\mathrm{with}\;\tilde{N}=1-\int dx\;\Psi_{x}^{*}\Psi_{x},$$

and parametrized by α is given by the vector field

(110) N¯=−Ψx/iℏiℏ(Ψx/iℏ)*.

Its integral curves are

(111) $$\Psi_{x}\left(\alpha \right)=\Psi_{x}\left(0\right)e^{i\alpha /\hbar }.$$

The constraint $\tilde{N}=0$ induces a gauge symmetry which leads us to restrict our attention to vectors $\bar{V}=d/d\lambda$ satisfying two real TGF conditions (83). In Ψ coordinates this is replaced by the single complex TGF condition,

(112) $$\int dx\;\Psi_{x}^{*}\frac{d\Psi_{x}}{d\lambda }=0.$$

In Ψ coordinates the metric on $T^{*}P$, Equation (98), becomes

(113) $$\delta {\tilde{s}}^{2}=-2i\int dx\;\delta \Psi_{x}\delta i\hbar \Psi_{x}^{*}=\int dxdx^{\prime }G_{\mu x,\nu x^{\prime }}\;\delta \Psi^{\mu x}\delta \Psi^{\nu x^{\prime }},$$

where the metric tensor and its inverse are

(114) $$G_{\mu x,\nu x^{\prime }}=-i\delta_{xx^{\prime }}\left[\begin{array}{cc} 0 & 1\\ 1 & 0 \end{array}\right]\;\mathrm{and}\;G^{\mu x,\nu x^{\prime }}=i\delta_{xx^{\prime }}\left[\begin{array}{cc} 0 & 1\\ 1 & 0 \end{array}\right].$$

Finally, using $G^{\mu x,\nu x^{\prime }}$ to raise the first index of $\Omega_{\nu x^{\prime },\gamma x^{\prime \prime }}$ gives the Ψ components of the tensor *J*

(115) $$J^{\mu x}{}_{\gamma x^{\prime \prime }}\overset{\mathrm{def}}{=}-G^{\mu x,\nu x^{\prime }}\Omega_{\nu x^{\prime },\gamma x^{\prime \prime }}=\left[\begin{array}{cc} i & 0\\ 0 & -i \end{array}\right]\delta_{xx^{\prime }}.$$

## 7. Hamilton-Killing Flows

Our next goal will be to find those Hamiltonian flows $Q^{I}$ that also happen to preserve the metric tensor, i.e., we want $Q^{I}$ to be a Killing vector. The condition for $Q^{I}$ is

(116) $${\left({£}_{Q}G\right)}_{IJ}=Q^{K}{\tilde{\nabla }}_{K}G_{IJ}+G_{KJ}{\tilde{\nabla }}_{I}Q^{K}+G_{IK}{\tilde{\nabla }}_{J}Q^{K}=0.$$

In complex coordinates Equation (114) gives ${\tilde{\nabla }}_{K}G_{IJ}=0$, and the Killing equation simplifies to

(117) $${\left({£}_{Q}G\right)}_{IJ}=G_{KJ}{\tilde{\nabla }}_{I}Q^{K}+G_{IK}{\tilde{\nabla }}_{J}Q^{K}=0,$$

or

(118) $${\left({£}_{Q}G\right)}_{\mu x,\nu x^{\prime }}=-i\left[\begin{array}{cc} \frac{\delta Q^{2x^{\prime }}}{\delta \Psi_{x}}+\frac{\delta Q^{2x}}{\delta \Psi_{x^{\prime }}}\;; & \frac{\delta Q^{1x^{\prime }}}{\delta \Psi_{x}}+\frac{\delta Q^{2x}}{\delta i\hbar \Psi_{x^{\prime }}^{*}}\\ \frac{\delta Q^{2x^{\prime }}}{\delta i\hbar \Psi_{x}^{*}}+\frac{\delta Q^{1x}}{\delta \Psi_{x^{\prime }}}\;; & \frac{\delta Q^{1x^{\prime }}}{\delta i\hbar \Psi_{x}^{*}}+\frac{\delta Q^{1x}}{\delta i\hbar \Psi_{x^{\prime }}^{*}} \end{array}\right]=0.$$

If we further require that $Q^{I}$ be a Hamiltonian flow, ${£}_{Q}\Omega =0$, then we substitute

(119) $$Q^{1x}=\frac{\delta \tilde{Q}}{\delta i\hbar \Psi_{x}^{*}}\;\mathrm{and}\;Q^{2x}=-\frac{\delta \tilde{Q}}{\delta \Psi_{x}}$$

into (118) to get

(120) $$\frac{\delta^{2}\tilde{Q}}{\delta \Psi_{x}\delta \Psi_{x^{\prime }}}=0\;\mathrm{and}\;\frac{\delta^{2}\tilde{Q}}{\delta \Psi_{x}^{*}\delta \Psi_{x^{\prime }}^{*}}=0.$$

Therefore, to generate a flow that preserves both *G* and Ω the functional $\tilde{Q}\left[\Psi ,\Psi^{*}\right]$ must be *linear* in both Ψ and $\Psi^{*}$,

(121) $$\tilde{Q}\left[\Psi ,\Psi^{*}\right]=\int dxdx^{\prime }\;\Psi_{x}^{*}{\hat{Q}}_{xx^{\prime }}\Psi_{x^{\prime }},$$

where ${\hat{Q}}_{xx^{\prime }}$ is a possibly non-local kernel. The actual Hamilton–Killing flow is

(122) $$\begin{array}{ccc} \frac{d\Psi_{x}}{d\lambda } & = & Q^{1x}=\frac{\delta \tilde{Q}}{\delta i\hbar \Psi_{x}^{*}}=\frac{1}{i\hbar }\int dx^{\prime }\;{\hat{Q}}_{xx^{\prime }}\Psi_{x^{\prime }}, \end{array}$$

(123) $$\begin{array}{ccc} \frac{di\hbar \Psi_{x}^{*}}{d\lambda } & = & Q^{2x}=-\frac{\delta \tilde{Q}}{\delta \Psi_{x}}=-\int dx^{\prime }\;\Psi_{x^{\prime }}^{*}{\hat{Q}}_{xx^{\prime }}. \end{array}$$

Taking the complex conjugate of (122) and compared to (123), shows that the kernel ${\hat{Q}}_{xx^{\prime }}$ is Hermitian,

(124) $${\hat{Q}}_{xx^{\prime }}^{*}={\hat{Q}}_{x^{\prime }x},$$

and we can check that the corresponding Hamiltonian functionals $\tilde{Q}$ are real,

$$\tilde{Q}{\left[\Psi ,\Psi^{*}\right]}^{*}=\tilde{Q}\left[\Psi ,\Psi^{*}\right].$$

The Hamiltonian flows that might potentially be of interest are those that generate symmetry transformations. For example, the generator of translations is total momentum. Under a spatial displacement by $\varepsilon^{a}$, $g\left(x\right)\to g_{\varepsilon }\left(x\right)=g\left(x-\varepsilon \right)$, the change in f[ρ,Φ] is

(125) $$\delta_{\varepsilon }f\left[\rho ,\Phi \right]=\int dx\left(\frac{\delta f}{\delta \rho_{x}}\delta_{\varepsilon }\rho_{x}+\frac{\delta f}{\delta \Phi_{x}}\delta_{\varepsilon }\Phi_{x}\right)=\left\{f,{\tilde{P}}_{a}\varepsilon^{a}\right\}$$

where

(126) $${\tilde{P}}_{a}=\int dx\;\rho \sum_{n}\frac{\partial \Phi }{\partial x_{n}^{a}}=\int dx\;\rho \frac{\partial \Phi }{\partial X^{a}}$$

is interpreted as the expectation of the total momentum, and $X^{a}$ are the coordinates of the center of mass,

(127) $$X^{a}=\frac{1}{M}\sum_{n}m_{n}x_{n}^{a}.$$

In complex coordinates,

(128) $${\tilde{P}}_{a}=\int dx\;\Psi^{*}\left(\sum_{n}\frac{\hbar }{i}\frac{\partial }{\partial x_{n}^{a}}\right)\Psi =\int dx\;\Psi^{*}\left(\frac{\hbar }{i}\frac{\partial }{\partial X^{a}}\right)\Psi ,$$

and the corresponding kernel ${\hat{P}}_{axx^{\prime }}$ is

(129) $${\hat{P}}_{axx^{\prime }}=\delta_{xx^{\prime }}\sum_{n}\frac{\hbar }{i}\frac{\partial }{\partial x_{n}^{a}}=\delta_{xx^{\prime }}\frac{\hbar }{i}\frac{\partial }{\partial X^{a}}.$$

## 8. The E-Hamiltonian

In the previous sections we supplied the symplectic e-phase space $T^{*}P$ with a Riemannian metric and, as a welcome by-product, also with a complex structure. Then we showed that the condition for the simplest form of dynamics—one that preserves all the metric, symplectic, and complex structures—is a Hamilton–Killing flow generated by a Hamiltonian $\tilde{H}$ that is linear in both Ψ and $\Psi^{*}$,

(130) $$\tilde{H}\left[\Psi ,\Psi^{*}\right]=\int dxdx^{\prime }\;\Psi_{x}^{*}{\hat{H}}_{xx^{\prime }}\Psi_{x^{\prime }}.$$

The last ingredient in the construction of $\tilde{H}$ is that the e-Hamiltonian must agree with (50) to reproduce the entropic evolution of ρ given by the continuity Equation (49).

To proceed we use the identity

(131) $$\frac{1}{2}\rho m^{AB}\left(\partial_{A}\Phi -{\bar{A}}_{A}\right)\left(\partial_{B}\Phi -{\bar{A}}_{B}\right)=\frac{\hbar^{2}}{2}m^{AB}{\left(D_{A}\Psi \right)}^{*}D_{B}\Psi -\frac{\hbar^{2}}{8\rho^{2}}m^{AB}\partial_{A}\rho \partial_{B}\rho$$

where

(132) $$D_{A}=\partial_{A}-\frac{i}{\hbar }{\bar{A}}_{A}\;\mathrm{and}\;{\bar{A}}_{A}\left(x\right)=\hbar \beta_{n}A_{a}\left(x_{n}\right).$$

Rewriting $\tilde{H}\left[\rho ,\Phi \right]$ in (50) in terms of Ψ and $\Psi^{*}$ we get

(133) $$\tilde{H}\left[\Psi ,\Psi^{*}\right]=\int dx\left(\frac{-\hbar^{2}}{2}m^{AB}\Psi^{*}D_{A}D_{B}\Psi \right)+F^{\prime }\left[\rho \right].$$

where

(134) $$F^{\prime }\left[\rho \right]=F\left[\rho \right]-\frac{\hbar^{2}}{8\rho^{2}}m^{AB}\partial_{A}\rho \partial_{B}\rho .$$

According to (121) for $\tilde{H}\left[\Psi ,\Psi^{*}\right]$ to generate an HK flow we must impose that $F^{\prime }\left[\rho \right]$ be linear in both Ψ and $\Psi^{*}$,

(135) $$F^{\prime }\left[\rho \right]=\int dxdx^{\prime }\;\Psi_{x}^{*}{\hat{V}}_{xx^{\prime }}\Psi_{x^{\prime }}$$

for some Hermitian kernel ${\hat{V}}_{xx^{\prime }}$, but $F^{\prime }\left[\rho \right]$ must remain independent of Φ,

(136) $$\frac{\delta F^{\prime }\left[\rho \right]}{\delta \Phi_{x}}=0\;.$$

Substituting $\Psi =\rho^{1/2}e^{i\Phi /\hbar }$ into (135) and using ${\hat{V}}_{x^{\prime }x}^{*}={\hat{V}}_{xx^{\prime }}$ leads to

(137) δF′δΦx=2ℏρx1/2∫dx′ρx′1/2ImV^xx′e−iΦx−Φx′/ℏ=0

This equation must be satisfied for all choices of $\rho_{x^{\prime }}$, which implies

(138) ImV^xx′e−iΦx−Φx′/ℏ=0,

and also for all choices of $\Phi_{x}$ and $\Phi_{x^{\prime }}$. Therefore, the kernel ${\hat{V}}_{xx^{\prime }}$ must be local in *x*,

(139) $${\hat{V}}_{xx^{\prime }}=\delta_{xx^{\prime }}V_{x},$$

where $V_{x}=V\left(x\right)$ is some real function.

We conclude that the Hamiltonian that generates a Hamilton–Killing flow and agrees with the ED continuity equation must be of the form

(140) $$\tilde{H}\left[\Psi ,\Psi^{*}\right]=\int dx\Psi^{*}\left(-\frac{\hbar^{2}}{2}m^{AB}D_{A}D_{B}+V\left(x\right)\right)\Psi .$$

The evolution of Ψ is given by the Hamilton equation,

(141) $$\partial_{t}\Psi_{x}=\left\{\Psi_{x},\tilde{H}\right\}=\frac{\delta \tilde{H}}{\delta (i\hbar \Psi^{*}\left(x\right))},$$

which is the Schrödinger equation,

(142) $$i\hbar \partial_{t}\Psi =-\frac{\hbar^{2}}{2}m^{AB}D_{A}D_{B}\Psi +V\Psi .$$

In more standard notation it reads

(143) $$i\hbar \partial_{t}\Psi =\sum_{n}\frac{-\hbar^{2}}{2m_{n}}\delta^{ab}\left(\frac{\partial }{\partial x_{n}^{a}}-i\beta_{n}A_{a}\left(x_{n}\right)\right)\left(\frac{\partial }{\partial x_{n}^{b}}-i\beta_{n}A_{b}\left(x_{n}\right)\right)\Psi +V\Psi .$$

At this point we can finally provide the physical interpretation of the various constants introduced along the way. Since the Schrödinger Equation (143) is the tool we use to analyze experimental data we can identify *ℏ* with Planck’s constant, $m_{n}$ will be interpreted as the particles’ masses, and the $\beta_{n}$ are related to the particles’ electric charges $q_{n}$ by

(144) $$\beta_{n}=\frac{q_{n}}{\hbar c}.$$

For completeness we write the Hamiltonian in the (ρ,Φ) variables,

(145) $$\begin{array}{ccc} \tilde{H}\left[\rho ,\Phi \right] & = & \int d^{3N}x\;\rho \left[\sum_{n}\frac{\delta^{ab}}{2m_{n}}\left(\frac{\partial \Phi }{\partial x_{n}^{a}}-\frac{q_{n}}{c}A_{a}\left(x_{n}\right)\right)\left(\frac{\partial \Phi }{\partial x_{n}^{b}}-\frac{q_{n}}{c}A_{b}\left(x_{n}\right)\right)\right.\\ & & +\left.\sum_{n}\frac{\hbar^{2}}{8m_{n}}\frac{\delta^{ab}}{\rho^{2}}\frac{\partial \rho }{\partial x_{n}^{a}}\frac{\partial \rho }{\partial x_{n}^{b}}+V\left(x_{1}\ldots x_{n}\right)\right]. \end{array}$$

The Hamilton equations for ρ and Φ are the continuity equation (49),

(146) $$\partial_{t}\rho =\frac{\delta \tilde{H}}{\delta \Phi }=-\sum_{n}\frac{\partial }{\partial x_{n}^{a}}\left[\rho \frac{\delta^{ab}}{m_{n}}\left(\frac{\partial \Phi }{\partial x_{n}^{b}}-\frac{q_{n}}{c}A_{b}\left(x_{n}\right)\right)\right],$$

and the quantum analogue of the Hamilton-Jacobi equation,

(147) $$\begin{array}{ccc} \partial_{t}\Phi & = & -\frac{\delta \tilde{H}}{\delta \rho }=\sum_{n}\frac{-\delta^{ab}}{2m_{n}}\left(\frac{\partial \Phi }{\partial x_{n}^{a}}-\frac{q_{n}}{c}A_{a}\left(x_{n}\right)\right)\left(\frac{\partial \Phi }{\partial x_{n}^{b}}-\frac{q_{n}}{c}A_{b}\left(x_{n}\right)\right)\\ & & +\left.\sum_{n}\frac{\hbar^{2}}{2m_{n}}\frac{\delta^{ab}}{\rho^{1/2}}\frac{\partial^{2}\rho^{1/2}}{\partial x_{n}^{a}\partial x_{n}^{b}}-V\left(x_{1}\ldots x_{n}\right)\right]. \end{array}$$

To summarize: we have just shown that an ED that preserves both the symplectic and metric structures of the e-phase space $T^{*}P$ leads to a linear Schrödinger equation. In particular, such an ED reproduces the quantum potential in (147) with the correct coefficients $\hbar^{2}/2m_{n}$.

## 9. Entropic Time, Physical Time, and Time Reversal

Now that the dynamics has been fully developed we revisit the question of time. The derivation of laws of physics as examples of inference led us to introduce the notion of entropic time which includes assumptions about the concept of instant, of simultaneity, of ordering, and of duration. It is clear that entropic time is useful but is this the actual, real, “physical” time? The answer is yes. By deriving the Schrödinger equation (from which we can obtain the classical limit) we have shown that the *t* that appears in the laws of physics is entropic time. Since these are the equations that we routinely use to design and calibrate our clocks we conclude that *what clocks measure is entropic time*. No notion of time that is in any way deeper or more “physical” is needed. Most interestingly, the entropic model automatically includes an arrow of time.

The statement that the laws of physics are invariant under time reversal has nothing to do with particles travelling backwards in time. It is instead the assertion that the laws of physics exhibit a certain symmetry. For a classical system described by coordinates *q* and momenta *p* the symmetry is the statement that if $\left\{q_{t},p_{t}\right\}$ happens to be one solution of Hamilton’s equations then we can construct another solution $\left\{q_{t}^{T},p_{t}^{T}\right\}$ where

(148) $$q_{t}^{T}=q_{-t}\;\mathrm{and}\;p_{t}^{T}=-p_{-t},$$

but both solutions $\left\{q_{t},p_{t}\right\}$ and $\left\{q_{t}^{T},p_{t}^{T}\right\}$ describe evolution forward in time. An alternative statement of time reversibility is the following: if there is one trajectory of the system that takes it from state $\left\{q_{0},p_{0}\right\}$ at time $t_{0}$ to state $\left\{q_{1},p_{1}\right\}$ at the later time $t_{1}$, then there is another possible trajectory that takes the system from state $\left\{q_{1},-p_{1}\right\}$ at time $t_{0}$ to state $\left\{q_{0},-p_{0}\right\}$ at the later time $t_{1}$. The merit of this re-statement is that it makes clear that nothing needs to travel back in time. Indeed, rather than time reversal the symmetry might be more appropriately described as momentum or motion reversal.

Since ED is a Hamiltonian dynamics one can expect that similar considerations will apply to QM and indeed they do. It is straightforward to check that given one solution $\left\{\rho_{t}\left(x\right),\Phi_{t}\left(x\right)\right\}$ that evolves forward in time, we can construct another solution $\left\{\rho_{t}^{T}\left(x\right),\Phi_{t}^{T}\left(x\right)\right\}$ that is also evolving forward in time. The reversed solution is

(149) $$\rho_{t}^{T}\left(x\right)=\rho_{-t}\left(x\right)\;\mathrm{and}\;\Phi_{t}^{T}\left(x\right)=-\Phi_{-t}\left(x\right).$$

These transformations constitute a symmetry—e.g., the transformed $\Psi_{t}^{T}\left(x\right)$ is a solution of the Schrödinger equation— provided the motion of the sources of the external potentials is also reversed, i.e., the potentials $A_{a}\left(\vec{x},t\right)$ and V(x,t) are transformed according to

(150) $$A_{a}^{T}\left(\vec{x},t\right)=-A_{a}\left(\vec{x},-t\right)\;\mathrm{and}\;V^{T}\left(x,t\right)=V\left(x,-t\right).$$

Expressed in terms of wave functions the time reversal transformation is

(151) $$\Psi_{t}^{T}\left(x\right)=\Psi_{-t}^{*}\left(x\right).$$

The proof that this is a symmetry is straightforward; just take the complex conjugate of (143), and let t→−t.

## 10. Linearity and the Superposition Principle

The Schrödinger equation is linear, i.e., a linear combination of solutions is a solution too. However, this *mathematical* linearity does not guarantee the *physical* linearity that is usually referred to as the superposition principle. The latter is the physical assumption that if there is one experimental setup that prepares a system in the (epistemic) state $\Psi_{1}$ and there is another setup that prepares the system in the state $\Psi_{2}$ then, at least in principle, it is possible to construct yet a third setup that can prepare the system in the superposition

(152) $$\Psi_{3}=\alpha_{1}\Psi_{1}+\alpha_{2}\Psi_{2},$$

where $\alpha_{1}$ and $\alpha_{2}$ are arbitrary complex numbers. Mathematical linearity refers to the fact that solutions can be expressed as sums of solutions. There is no implication that any of these solutions will necessarily describe physical situations. Physical linearity on the other hand—the Superposition Principle—refers to the fact that the superposition of physical solutions is also a physical solution. The point to be emphasized is the Superposition Principle is not a principle; it is a physical hypothesis that need not be universally true.

### 10.1. The Single-Valuedness of *Ψ*

The question “Why should wave functions be single-valued?” has been around for a long time. In this section we build on and extend recent work [100] to argue that the single- or multi-valuedness of the wave functions is closely related to the question of linearity and the superposition principle. Our discussion parallels that by Schrödinger [101,102]. (See also [103,104,105,106,107,108,109,110].)

To show that the mathematical linearity of (143) is not sufficient to imply the superposition principle, we argue that even when $|\Psi_{1}{|}^{2}=\rho_{1}$ and $|\Psi_{2}{|}^{2}=\rho_{2}$ are probabilities it is not generally true that $|\Psi_{3}{|}^{2}$, Equation (152), will also be a probability. Consider moving around a closed loop Γ in configuration space. Since phases Φ(x) can be multi-valued the corresponding wave functions could in principle be multi-valued too. Let a generic Ψ change by a phase factor,

(153) $$\Psi \to \Psi^{\prime }=e^{i\delta }\Psi ,$$

then the superposition $\Psi_{3}$ of two wave functions $\Psi_{1}$ and $\Psi_{2}$ changes into

(154) $$\Psi_{3}\to \Psi_{3}^{\prime }=\alpha_{1}e^{i\delta_{1}}\Psi_{1}+\alpha_{2}e^{i\delta_{2}}\Psi_{2}.$$

The problem is that even if $|\Psi_{1}{|}^{2}=\rho_{1}$ and $|\Psi_{2}{|}^{2}=\rho_{2}$ are single-valued (because they are probability densities), the quantity $|\Psi_{3}{|}^{2}$ need not in general be single-valued. Indeed,

(155) $$|\Psi_{3}{|}^{2}=|\alpha_{1}{|}^{2}\rho_{1}+{\left|\alpha_{2}\right|}^{2}\rho_{2}+2Re\left[\alpha_{1}\alpha_{2}^{*}\Psi_{1}\Psi_{2}^{*}\right],$$

changes into

(156) $$|\Psi_{3}^{\prime }{|}^{2}=|\alpha_{1}{|}^{2}\rho_{1}+{\left|\alpha_{2}\right|}^{2}\rho_{2}+2Re\left[\alpha_{1}\alpha_{2}^{*}e^{i(\delta_{1}-\delta_{2})}\Psi_{1}\Psi_{2}^{*}\right],$$

so that in general

(157) $$|\Psi_{3}^{\prime }{|}^{2}\neq {\left|\Psi_{3}\right|}^{2},$$

which precludes the interpretation of $|\Psi_{3}{|}^{2}$ as a probability. That is, even when the epistemic states $\Psi_{1}$ and $\Psi_{2}$ describe actual physical situations, their superpositions need not.

The problem does not arise when

(158) $$e^{i(\delta_{1}-\delta_{2})}=1.$$

If we were to group the wave functions into classes each characterized by its own δ then we could have a limited version of the superposition principle that applies within each class. We conclude that beyond the linearity of the Schrödinger equation we have a superselection rule that restricts the validity of the superposition principle to wave functions belong to the same δ-class.

To find the allowed values of δ we argue as follows. It is natural to assume that if {ρ,Φ} (at some given time $t_{0}$) is a physical state then the state with reversed momentum {ρ,−Φ} (at the same time $t_{0}$) is an equally reasonable physical state. Basically, the idea is that if particles can be prepared to move in one direction, then they can also be prepared to move in the opposite direction. In terms of wave functions the statement is that if $\Psi_{t_{0}}$ is a physically allowed initial state, then so is $\Psi_{t_{0}}^{*}$ [111]. Next we consider a generic superposition

(159) $$\Psi_{3}=\alpha_{1}\Psi +\alpha_{2}\Psi^{*}.$$

Is it physically possible to construct superpositions such as (159)? The answer is that while constructing $\Psi_{3}$ for an arbitrary Ψ might not be feasible in practice there is strong empirical evidence that there exist no superselection rules to prevent us from doing so in principle. Indeed, it is easy to construct superpositions of wavepackets with momentum $\vec{p}$ and $-\vec{p}$, or superpositions of states with opposite angular momenta, $Y_{\ell m}$ and $Y_{\ell ,-m}$. *We shall assume that in principle the superpositions (159) are physically possible.*

According to Equation (153) as one moves in a closed loop Γ the wave function $\Psi_{3}$ will transform into

(160) $$\Psi_{3}^{\prime }=\alpha_{1}e^{i\delta }\Psi +\alpha_{2}e^{-i\delta }\Psi^{*},$$

and the condition (158) for $|\Psi_{3}{|}^{2}$ to be single-valued is

(161) $$e^{2i\delta }=1\;\mathrm{or}\;e^{i\delta }=\pm 1.$$

Thus, we are restricted to two discrete possibilities ±1. Since the wave functions are assumed sufficiently well behaved (continuous, differentiable, etc.) we conclude that they must be either single-valued, $e^{i\delta }=1$, or double-valued, $e^{i\delta }=-1$.

We conclude that the Superposition Principle appears to be valid in a sufficiently large number of cases to be a useful rule of thumb but it is restricted to single-valued (or double-valued) wave functions. The argument above does not exclude the possibility that a multi-valued wave function might describe an actual physical situation. What the argument implies is that the Superposition Principle would not extend to such states.

### 10.2. Charge Quantization

Next we analyze the conditions for the electromagnetic gauge symmetry to be compatible with the superposition principle. We shall confine our attention to systems that are described by single-valued wave functions ($e^{i\delta }=+1$) [112]. The condition for the wave function to be single-valued is

(162) $$\Delta \frac{\Phi }{\hbar }=\oint_{\Gamma }d\ell^{A}\partial_{A}\frac{\Phi }{\hbar }=2\pi k_{\Gamma }\;,$$

where $k_{\Gamma }$ is an integer that depends on the loop Γ. Under a local gauge transformation

(163) $$A_{a}\left(\vec{x}\right)\to A_{a}\left(\vec{x}\right)+\partial_{a}\chi \left(\vec{x}\right)$$

the phase Φ transforms according to (38),

(164) $$\Phi \left(x\right)\to \Phi^{\prime }\left(x\right)=\Phi \left(x\right)+\sum_{n}\frac{q_{n}}{c}\chi \left({\vec{x}}_{n}\right).$$

The requirement that the gauge symmetry and the superposition principle be compatible amounts to requiring that the gauge transformed states also be single-valued,

(165) $$\Delta \frac{\Phi^{\prime }}{\hbar }=\oint_{\Gamma }d\ell^{A}\partial_{A}\frac{\Phi^{\prime }}{\hbar }=2\pi k_{\Gamma }^{\prime }.$$

Thus, the allowed gauge transformations are restricted to functions $\chi (\vec{x})$ such that

(166) $$\sum_{n}\frac{q_{n}}{\hbar c}\oint_{\Gamma }d\ell_{n}^{a}\partial_{na}\chi \left({\vec{x}}_{n}\right)=2\pi \Delta k_{\Gamma }$$

where $\Delta k_{\Gamma }=k_{\Gamma }^{\prime }-k_{\Gamma }$ is an integer. Consider now a loop γ in which we follow the coordinates of the *n*th particle around some closed path in 3-dimensional space while all the other particles are kept fixed. Then

(167) $$\frac{q_{n}}{\hbar c}\oint_{\gamma }d\ell_{n}^{a}\partial_{an}\chi \left({\vec{x}}_{n}\right)=2\pi \Delta k_{n\gamma }$$

where $\Delta k_{n\gamma }$ is an integer. Since the gauge function $\chi (\vec{x})$ is just a function in 3-dimensional space it is the same for all particles and the integral on the left is independent of *n*. This implies that the charge $q_{n}$ divided by an integer $\Delta k_{n\gamma }$ must be independent of *n* which means that $q_{n}$ must be an integer multiple of some basic charge $q_{0}$. We conclude that the charges $q_{n}$ are quantized.

The issue of charge quantization is ultimately the issue of deciding which is the gauge group that generates electromagnetic interactions. We could for example decide to restrict the gauge transformations to single-valued gauge functions $\chi (\vec{x})$ so that (167) is trivially satisfied irrespective of the charges being quantized or not. Under such a restricted symmetry group the single-valued (or double-valued) nature of the wave function is unaffected by gauge transformations. If, on the other hand, the gauge functions $\chi (\vec{x})$ are allowed to be multi-valued, then the compatibility of the gauge transformation (163)–(164) with the superposition principle demands that charges be quantized.

The argument above cannot fix the value of the basic charge $q_{0}$ because it depends on the units chosen for the vector potential $A_{a}$. Indeed since the dynamical equations show $q_{n}$ and $A_{a}$ appearing only in the combination $q_{n}A_{a}$ we can change units by rescaling charges and potentials according to $Cq_{n}=q_{n}^{\prime }$ and $A_{a}/C=A_{a}^{\prime }$ so that $q_{n}A_{a}=q_{n}^{\prime }A_{a}^{\prime }$. For conventional units such that the basic charge is $q_{0}=e/3$ with $\alpha =e^{2}/\hbar c=1/137$ the scaling factor is $C={\left(\alpha \hbar c\right)}^{1/2}/3q_{0}$. A more natural set of units might be to set $q_{0}=\hbar c$ so that all $\beta_{n}$s are integers and the gauge functions $\chi (\vec{x})$ are angles.

A similar conclusion—that charge quantization is a reflection of the compactness of the gauge group—can be reached following an argument due to C. N. Yang [113]. Yang’s argument assumes that a Hilbert space has been established and one has access to the unitary representations of symmetry groups. Yang considers a gauge transformation

(168) $$\Psi \left(x\right)\to \Psi \left(x\right)\exp i\sum_{n}\frac{q_{n}}{c}\chi \left({\vec{x}}_{n}\right),$$

with $\chi (\vec{x})$ independent of $\vec{x}$. If the $q_{n}$s are not commensurate there is no value of χ (except 0) that makes (168) be the identity transformation. The gauge group—translations on the real line—would not be compact. If, on the other hand, the charges are integer multiples of a basic charge $q_{0}$, then two values of χ that differ by an integer multiple of $2\pi c/q_{0}$ give identical transformations and the gauge group is compact. In the present ED derivation, however, we deal with the space $T^{*}P$ which is a complex projective space. We cannot adopt Yang’s argument because a gauge transformation χ independent of $\vec{x}$ is already an identity transformation—it leads to an equivalent state in the same ray—and cannot therefore lead to any constraints on the allowed charges.

## 11. The Classical Limits and the Bohmian Limit

### 11.1. Classical Limits

There are two classical limits that one might wish to consider. One is the mathematical limit ℏ→0. Taking ℏ→0 leaves unchanged both the velocities $v_{n}^{a}$ of the particles, Equation (19), and the probability flow, Equation (146). The main effect is to suppress the quantum potential so that Equation (147) becomes the classical Hamilton-Jacobi equation. The symplectic form, Equation (63), survives unscathed but the metric and the complex structures, Equations (101) and (103), do not. However, this is not quite classical mechanics. Since the velocity fluctuations, Equation (25), remain unaffected the resulting dynamics is a non-dissipative version of the classical Oernstein–Uhlenbeck Brownian motion. To recover a deterministic classical mechanics one must also take the limit η→0.

The other classical limit arises in the more physically relevant situation where one deals with a system with a large number *N* of particles—for example, a speck of dust—and one wishes to study the motion of an effective macrovariable such as the center of mass (CM), Equation (127). The large *N* limit of ED with particles undergoing an ES Brownian motion was studied in [77]. The same argument goes through essentially unchanged for the OU Brownian motion discussed here. Skipping all details we find that because of the central limit theorem the continuity equation for $\rho_{\mathrm{cm}}\left(X^{a}\right)$ and the velocity fluctuations are given by the analogues of (43) and (25) for a single particle of mass $M=\sum_{n=1}^{N}m_{n}$,

(169) $$\partial_{t}\rho_{\mathrm{cm}}=\frac{\partial }{\partial X^{a}}\left(\rho_{\mathrm{cm}}V^{a}\right)\;\mathrm{with}\;V^{a}=\frac{\langle \Delta X^{a}\rangle }{\Delta t}=\frac{1}{M}\frac{\partial \Phi_{\mathrm{cm}}}{\partial X^{a}},$$

(170) $$\langle \left(\frac{\Delta X^{a}}{\Delta t}-V^{a}\right)\left(\frac{\Delta X^{b}}{\Delta t}-V^{b}\right)\rangle =\frac{\eta \Delta t}{M}.$$

We also find that under rather general conditions the CM motion decouples from the motion of the component particles and obeys the single particle HJ equation

(171) $$-\partial_{t}\Phi_{\mathrm{cm}}=\frac{1}{2M}{\left(\frac{\partial \Phi_{\mathrm{cm}}}{\partial X^{a}}\right)}^{2}-\frac{\hbar^{2}}{2M}\frac{\nabla^{2}\rho_{\mathrm{cm}}^{1/2}}{\rho_{\mathrm{cm}}^{1/2}}+V_{\mathrm{ext}}\left(X\right).$$

In the large *N* limit M∼O(N) and we obtain a finite velocity $V^{a}$ in (169) provided $\Phi_{\mathrm{cm}}∼O\left(N\right)$. In Equation (171) we see that for a sufficiently large system the quantum potential for the CM motion vanishes. Therefore, for N→∞, the CM follows smooth trajectories described by a classical Hamilton-Jacobi equation. Furthermore, Equation (170) shows that as N→∞ the velocity fluctuations vanish irrespective of the value of η. This is a truly deterministic classical mechanics.

An important feature of this derivation is that *ℏ* and η remain finite which means that a mesoscopic or macroscopic object will behave classically while all its component particles remain fully quantum mechanical.

### 11.2. The Bohmian Limit

ED models with different values of η lead to the same Schrödinger equation. In other words, different sub-quantum models lead to the same emergent quantum behavior. The limit of vanishing η deserves particular attention because the velocity fluctuations, Equation (25), are suppressed and the motion becomes deterministic. This means that ED includes the Bohmian form of quantum mechanics [51,52,53] as a special limiting case—but with the important caveat that the difference in physical interpretation remains enormous. It is only with respect to the mathematical formalism that ED includes Bohmian mechanics as a special case.

Bohmian mechanics attempts to provide an actual description of reality. In the Bohmian view the universe consists of real particles that have definite positions and their trajectories are guided by a real field, the wave function Ψ. Not only does this pilot wave live in 3N-dimensional configuration space but it manages to act on the particles without the particles reacting back upon it. These are peculiarities that have stood in the way of a wider acceptance of the Bohmian interpretation. In contrast, ED’s pragmatic goal is much less ambitious: to make the best possible predictions based on very incomplete information. As in Bohmian mechanics, in ED the particles also have definite positions and its formalism includes a function Φ that plays the role of a pilot wave. However, Φ is an epistemic tool for reasoning; it is not meant to represent anything real. There is no implication that the particles move the way they do because they are pushed around by a pilot wave or by some stochastic force. In fact, ED is silent on the issue of what if anything is pushing the particles. What the probability ρ and the phase Φ are designed to do is not to guide the particles but to guide our inferences. They guide our expectations of where and when to find the particles but they do not exert any causal influence on the particles themselves.

## 12. Hilbert Space

The formulation of the ED of spinless particles is now complete. We note, in particular, that the notion of Hilbert spaces turned out to be unnecessary to the formulation of quantum mechanics. As we shall see next, while strictly unnecessary in principle, the introduction of Hilbert spaces is nevertheless very convenient for calculational purposes.

**A vector space**. As we saw above the infinite-dimensional e-phase space—the cotangent bundle $T^{*}P$—is difficult to handle. The problem is that the natural coordinates are probabilities $\rho_{x}$ which, due to the normalization constraint, are not independent. In a discrete space one could single out one of the coordinates and its conjugate momentum and then proceed to remove them. Unfortunately, with a continuum of coordinates and momenta the removal is not feasible. The solution is to embed $T^{*}P$ in a larger space $T^{*}P^{+1}$. This move allows us to keep the natural coordinates $\rho_{x}$ but there is a price: we are forced to deal with a constrained system and its attendant gauge symmetry.

We also saw that the geometry of the embedding space was not fully determined: any spherically symmetric space would serve our purposes. This is a freedom we can further exploit. For calculational purposes the linearity of the Schrödinger Equation (143) is very convenient but its usefulness is severely limited by the normalization constraint. If $\Psi_{1}$ and $\Psi_{2}$ are flows in $T^{*}P$ then the superposition $\Psi_{3}$ in (152) will also be a flow in $T^{*}P$ but only if the coefficients $\alpha_{1}$ and $\alpha_{2}$ are such that $\Psi_{3}$ is properly normalized. This restriction can be removed by choosing the extended embedding space $T^{*}P^{+1}$ to be flat—just set A=0 and B=1 in Equation (91). (The fact that this space is flat is evident in the metric (89) for the discrete case.) We emphasize that this choice is not at all obligatory; it is optional.

The fact that in the flat space $T^{*}P^{+1}$ superpositions are allowed for arbitrary constants $\alpha_{1}$ and $\alpha_{2}$ means that $T^{*}P^{+1}$ is not just a manifold; it is also a vector space. Each point Ψ in $T^{*}P^{+1}$ is itself a vector. Furthermore, since the vector tangent to a curve is just a difference of two vectors Ψ we see that that points on the manifold and vectors tangent to the manifold are objects of the same kind. In other words, the tangent spaces $T{\left[T^{*}P^{+1}\right]}_{\Psi }$ are identical to the space $T^{*}P^{+1}$ itself.

The symplectic form Ω and the metric tensor *G* on the extended space $T^{*}P^{+1}$ are given by Equations (108) and (114). Since they are tensors Ω and *G* are meant to act on vectors but now they can also act on all points $\Psi \in T^{*}P^{+1}$ and not just on those that happen to be normalized and gauge fixed according to (83). For example, the action of the mixed tensor *J*, Equation (115), on a wave function Ψ is

(172) Jμxνx′Ψνx′=i00−iΨxiℏΨx*=iΨxiℏ(iΨx)*,

which indicates that *J* plays the role of multiplication by *i*, i.e., when acting on a point Ψ the action of *J* is $\Psi \overset{J}{\to }i\Psi$.

**Dirac notation**. We can at this point introduce the Dirac notation to represent the wave functions $\Psi_{x}$ as vectors |Ψ〉 in a Hilbert space. The scalar product $\langle \Psi_{1}|\Psi_{2}\rangle$ is defined using the metric *G* and the symplectic form Ω,

(173) 〈Ψ1|Ψ2〉=def12ℏ∫dxdx′Ψ1x,iℏΨ1x*G+iΩΨ2x′iℏΨ2x′*.

A straightforward calculation gives

(174) $$\langle \Psi_{1}|\Psi_{2}\rangle =\int dx\;\Psi_{1}^{*}\Psi_{2}.$$

The map $\Psi_{x}↔|\Psi \rangle$ is defined by

(175) $$|\Psi \rangle =\int dx|x\rangle \Psi_{x}\;\mathrm{where}\;\Psi_{x}=\langle x|\Psi \rangle ,$$

where, in this “position” representation, the vectors {|x〉} form a basis that is orthogonal and complete,

(176) $$\langle x|x^{\prime }\rangle =\delta_{xx^{\prime }}\;\mathrm{and}\;\int dx\;|x\rangle \langle x|=\hat{1}.$$

**Hermitian and unitary operators**. The bilinear Hamilton functionals $\tilde{Q}\left[\Psi ,\Psi^{*}\right]$ with kernel $\hat{Q}\left(x,x^{\prime }\right)$ in Equation (121) can now be written in terms of a Hermitian operator $\hat{Q}$ and its matrix elements,

(177) $$\tilde{Q}\left[\Psi ,\Psi^{*}\right]=\langle \Psi |\hat{Q}|\Psi \rangle \;\mathrm{and}\;\hat{Q}\left(x,x^{\prime }\right)=\langle x|\hat{Q}|x^{\prime }\rangle .$$

The corresponding Hamilton–Killing flows are given by

(178) $$i\hbar \frac{d}{d\lambda }\langle x|\Psi \rangle =\langle x|\hat{Q}|\Psi \rangle \;\mathrm{or}\;i\hbar \frac{d}{d\lambda }|\Psi \rangle =\hat{Q}|\Psi \rangle .$$

These flows are described by unitary transformations

(179) $$|\Psi \left(\lambda \right)\rangle ={\hat{U}}_{Q}\left(\lambda \right)|\Psi \left(0\right)\rangle \;\mathrm{where}\;{\hat{U}}_{Q}\left(\lambda \right)=\exp\left(-\frac{i}{\hbar }\hat{Q}\lambda \right).$$

**Commutators**. The Poisson bracket of two Hamiltonian functionals $\tilde{U}\left[\Psi ,\Psi^{*}\right]$ and $\tilde{V}\left[\Psi ,\Psi^{*}\right]$,

$$\left\{\tilde{U},\tilde{V}\right\}=\int dx\left(\frac{\delta \tilde{U}}{\delta \Psi_{x}}\frac{\delta \tilde{V}}{\delta i\hbar \Psi_{x}^{*}}-\frac{\delta \tilde{U}}{\delta i\hbar \Psi_{x}^{*}}\frac{\delta \tilde{V}}{\delta \Psi_{x}}\right),$$

can be written in terms of the commutator of the associated operators, then

(180) $$\left\{\tilde{U},\tilde{V}\right\}=\frac{1}{i\hbar }\langle \Psi |\left[\hat{U},\hat{V}\right]|\Psi \rangle .$$

Thus, the Poisson bracket is the expectation of the commutator. This *identity* is much sharper than Dirac’s pioneering discovery that the quantum commutator of two *q*-variables is *analogous* to the Poisson bracket of the corresponding classical variables. Further parallels between the geometric and the Hilbert space formulation of QM can be found in [56,57,58,59,60,61,62,63,64].

## 13. Remarks on ED and Quantum Bayesianism

Having discussed the ED approach in some detail it is now appropriate to comment on how ED differs from the interpretations known as Quantum Bayesianism [20,21,22] and its closely related descendant QBism [23,24]; for simplicity, I shall refer to both as QB. Both ED and QB adopt an epistemic degree-of-belief concept of probability but there are important differences:

(a) QB adopts a personalistic de Finetti type of Bayesian interpretation while ED adopts an impersonal entropic Bayesian interpretation somewhat closer but not identical to Jaynes’ [15,16,17,18]. In ED, the probabilities do not reflect the subjective beliefs of any particular person. They are tools designed to assist us in those all too common situations in which are confused and due to insufficient information we do not know what to believe. The probabilities will then provide guidance as to what agents ought to believe if only they were ideally rational. More explicitly, probabilities in ED describe the objective degrees of belief of ideally rational agents who have been supplied with the maximal allowed information about a particular quantum system.
(b) ED derives or reconstructs the mathematical framework of QM—it explains where the symplectic, metric, and complex structures, including Hilbert spaces and time evolution come from. In contrast, at its current stage of development QB consists of appending a Bayesian interpretation to an already existing mathematical framework. Indeed, assumptions and concepts from quantum information are central to QB and are implicitly adopted from the start. For example, a major QB concern is the justification of the Born rule starting from the Hilbert space framework while ED starts from probabilities and its goal is to justify the construction of wave functions; the Born rule follows as a trivial consequence.
(c) ED is an application of entropic/Bayesian inference. Of course, the choices of variables and of the constraints that happen to be physically relevant are specific to our subject matter—quantum mechanics—but the inference method itself is of universal applicability. It applies to electrons just as well as to the stock market or to medical trials. In contrast, in QB the personalistic Bayesian framework is not of universal validity. For those special systems that we call ‘quantum’ the inference framework is itself modified into a new “Quantum-Bayesian coherence” in which the standard Bayesian inference must be supplemented with concepts from quantum information theory. The additional technical ingredient is a hypothetical structure called a “symmetric informationally complete positive-operator-valued measure”. In short, in QB Born’s Rule is not derived but constitutes an addition beyond the raw probability theory.
(d) QB is an anti-realist neo-Copenhagen interpretation; it accepts complementarity. (Here complementarity is taken to be the common thread that runs through all Copenhagen interpretations.) Probabilities in QB refer to the outcomes of experiments and not to ontic pre-existing values. In contrast, in ED probabilities refer to ontic positions—including the ontic positions of pointer variables. In the end, this is what solves the problem of quantum measurement (see [70,71]).

## 14. Some Final Remarks

We conclude with a summary of the main assumptions:

- Particles have definite but unknown positions and follow continuous trajectories.
- The probability of a short step is given by the method of maximum entropy subject to a drift potential constraint that introduces directionality and correlations, plus gauge constraints that account for external electromagnetic fields.
- The accumulation of short steps requires a notion of time as a book-keeping device. This involves the introduction of the concept of an instant and a convenient definition of the duration between successive instants.
- The e-phase space {ρ,Φ} has a natural symplectic geometry that results from treating the pair $\left(\rho_{x},\Phi_{x}\right)$ as canonically conjugate variables.
- The information geometry of the space of probabilities is extended to the full e-phase space by imposing the latter be spherically symmetric.
- The drift potential constraint is updated instant by instant in such a way as to preserve both the symplectic and metric geometries of the e-phase space.

The resulting entropic dynamics is described by the Schrödinger equation. Different sub-quantum Brownian motions all lead to the same emergent quantum mechanics. In previous work we dealt with an Einstein–Smoluchowski process; here we have explored an Oernstein–Uhlenbeck process. Other “fractional” Brownian motions might be possible but have not yet been studied.

A natural question is whether these different sub-quantum Brownian motions might have observable consequences. At this point our answer can only be tentative. To the extent that we have succeeded in deriving QM and not some other theory one should not expect deviations in the predictions for the standard experiments that are the subject of the standard quantum theory— at least not in the non-relativistic regime. As the ED program is extended to other regimes involving higher energies and/or gravity it is quite possible that those different sub-quantum motions might not be empirically equivalent.

ED achieves ontological clarity by sharply separating the ontic elements from the epistemic elements — positions of particles on one side and probabilities ρ and phases Φ on the other. ED is a dynamics of probabilities and not a dynamics of particles. Of course, if probabilities at one instant are large in one place and at a later time they are large in some other place one infers that the particles must have moved—but nothing in ED describes what it is that has pushed the particles around. ED is a mechanics without a mechanism.

We can elaborate on this point from a different direction. The empirical success of ED suggests that its epistemic probabilities agree with ontic features of the physical world. It is highly desirable to clarify the precise nature of this agreement. Consider, for example, a fair die. Its property of being a perfect cube is an ontic property of the die which is reflected at the epistemic level in the equal assignment of probabilities to each face of the die. In this example we see that the epistemic probabilities achieve objectivity, and therefore usefulness, by corresponding to something ontic. The situation in ED is similar except for one crucial aspect. The ED probabilities are objective, and they are empirically successful. They must therefore reflect something real. However, it is not yet known what those underlying ontic properties might possibly be. Fortunately, for the purposes of making predictions knowing those epistemic probabilities is all we need.

The trick of embedding the e-phase space $T^{*}P$ in a *flat* vector space $T^{*}P^{+1}$ is clever but optional. It allows one to make use of the calculational advantages of linearity. This recognition that Hilbert spaces are not fundamental is one of the significant contributions of the entropic approach to our understanding of QM. The distinction—whether Hilbert spaces are necessary in principle as opposed to merely convenient in practice—is not of purely academic interest. It can be important in the search for a quantum theory that includes gravity: Shall we follow the usual approaches to quantization that proceed by replacing classical dynamical variables by an algebra of linear operators acting on some abstract space? Or, in the spirit of an entropic dynamics, shall we search for an appropriately constrained dynamics of probabilities and information geometries? First steps towards formulating a first-principles theory along these lines are given in [114,115].
